# Non-use Economic Values for Little-Known Aquatic Species at Risk: Comparing Choice Experiment Results from Surveys Focused on Species, Guilds, and Ecosystems

**DOI:** 10.1007/s00267-016-0716-0

**Published:** 2016-06-13

**Authors:** Murray A. Rudd, Sheri Andres, Mary Kilfoil

**Affiliations:** 1 Department of Environmental Sciences, Emory University, Atlanta, GA USA; 2 Fisheries and Oceans Canada, Winnipeg, MB Canada; 3 Gardner Pinfold Consulting Economists Ltd, Halifax, NS Canada; 4 Rowe School of Business, Faculty of Management, Dalhousie University, Halifax, NS Canada

**Keywords:** River, Wetland, Endangered species, Ecosystem services, Ecosystem approach, Fish

## Abstract

Accounting for non-market economic values of biological diversity is important to fully assess the benefits of environmental policies and regulations. This study used three choice experiments (species-, guild-, and ecosystem-based surveys) in parallel to quantify non-use values for little-known aquatic species at risk in southern Ontario. Mean willingness-to-pay (WTP) ranged from $9.45 to $21.41 per listing status increment under Canada’s *Species at Risk Act* for both named and unnamed little-known species. Given the broad range of valuable ecosystem services likely to accrue to residents from substantial increases in water quality and the rehabilitation of coastal wetlands, the difference in WTP between species- and ecosystem-based surveys seemed implausibly small. It appeared that naming species—the ‘iconization’ of species in two of the three surveys—had an important effect on WTP. The results suggest that reasonable annual household-level WTP values for little-known aquatic species may be $10 to $25 per species or $10 to $20 per listing status increment. The results highlighted the utility of using parallel surveys to triangulate on non-use economic values for little-known species at risk.

**Electronic supplementary material:**

The online version of this article (doi:10.1007/s00267-016-0716-0) contains supplementary material, which is available to authorized users.

## Introduction

Economic valuation of final ecosystem services, biophysical outcomes which directly enhance the welfare of human beneficiaries (Fisher et al. 2009; Johnston and Russell 2011), is recognized as an integral part of the ecosystem services approach to environmental management (Bateman et al. 2011). Specification of the benefits of ecosystem services is, however, complicated because changes in ecological (e.g., species, populations, habitats) and environmental (e.g., water quality and flow) factors and attributes can provide multiple benefit flows for different people and across regions. The economic valuation of ecosystem service benefits is also challenging because of the absence of markets for these goods, but stated preference surveys now provide an approach for estimating the inferred value that citizens hold for ecosystem services. Choice experiments, one type of stated preference survey, have been widely used to value non-use environmental benefits now for 20 years (Adamowicz et al. 1998; Hanley et al. 2001).

The total economic value of an ecosystem service consists of both non-use and use values (which people derive from consumption, recreation, etc.). For little-known species, use values may be negligible, and non-use values, which arise as a result of citizens’ simply knowing that species exist or will survive into the future (Pearce and Moran 1994), may comprise much or all of the economic value of those species (e.g., Veisten et al. 2004; Jacobsen et al. 2007; Rudd 2009; Johnston et al. 2011; Lew 2015). While non-use values may be low for individuals, the potentially broad geographic scope across which citizens derive benefits means that those species can still be the source of very substantial aggregate non-use values at national (e.g., Loomis 2000; Rudd 2009; Lew and Wallmo 2011) or international levels (e.g., Hein et al. 2006).

Most commonly, individual species at risk are often the focus of conservation efforts precisely because of their rarity and vulnerability to a host of human-induced threats. At least 36 nations now have species-oriented conservation legislation to identify and protect species at risk (Mooers et al. 2010). Each time a little-known species is protected it will incur costs, which must ultimately be paid by citizens. Given the substantive costs of quantifying non-use benefits for little-known species via stated preference surveys and the ecological similarity between suites of little-known species, one strategy for reducing the costs of assessing and protecting those species is to move from species-oriented to guild- (i.e., a group of similar species within a specific habitat) or ecosystem-oriented assessments and recovery initiatives. Valuation of ecosystem services at a broader ecological scale may be more economical (Richardson and Loomis 2009) but could also lead to confounding of multiple intermediate and final ecosystem services, thus reducing their utility for policy purposes. Another strategy to reduce the need for expensive primary valuation research is to use benefits transfer, the practice of using values derived in one location and context at other different locations in the future (Hanley et al. 2001; Johnston et al. 2005; Akter and Grafton 2010).

Three choice experiment surveys, commissioned in 2011 by Canada’s Department of Fisheries and Oceans (DFO), were used to quantify non-use values for little-known freshwater aquatic species at risk in southern Ontario. The goal was to assess whether little-known aquatic species at risk could be valued in such a way to provide useful information for future benefits transfer research and provide information about the feasibility of using guild- or ecosystem-level research to value individual species. Non-use values are recognized by the Government of Canada as a valid component of economic analysis (Treasury Board Secretariat 2007), and ecosystem service valuation was recently highlighted as a key Canadian conservation science research need (Rudd et al. 2011). The first survey focused on the valuation of three individual species proposed for listing under the *Species at Risk Act* (SARA), one of which is relatively well-known (lake sturgeon, *Acipenser fulvescens*) and two of which are little-known (pugnose shiner, *Notropis anogenus*; channel darter, *Percina copelandi*). Respondents were queried about their preferences for conservation interventions that led to changes in listing status for both pugnose shiner and channel darter, and for change in recovery time for lake sturgeon. Changes in listing status for the little-known species should only affect respondents’ non-use values for channel darter and pugnose shiner. Given the broader range of potential ecosystem services that lake sturgeon provides (i.e., recreational and commercial fishing opportunities, a potentially important ecological role as a large scavenger species), values derived for that species may represent multiple types of benefits.

The second survey focused on the valuation of changes in SARA listing status for species within species guilds, groups of riverine (channel darter; eastern sand darter, *Ammocrypta pellucida*; and spotted sucker, *Minytrema melanops*) and coastal freshwater wetland (pugnose shiner; lake chubsucker, *Erimyzon sucetta*; spotted gar, *Lepisosteus oculatus*; pugnose minnow, *Opsopoeodus emiliae*; and warmouth, *Lepomis gulosus*) species. While listing status can here be considered an indicator of non-use value arising from protection of little-known species, the survey instrument specifically used ‘improved water quality’ and ‘securing and rehabilitating coastal wetlands’ as the means by which improvements in guild species would be secured. Thus there was potential for survey respondents to consider some broader ecosystem services beyond the benefits accruing solely to changes in the listing status of guild members.

The third ecosystem-oriented survey focused broadly on investments to improve water quality, and to secure and rehabilitate coastal wetlands. The survey specified that listing status improvements for unnamed aquatic species at risk would result, and that improvements in water quality, and securing and rehabilitating coastal wetlands, would have other broad impacts beyond species at risk recovery. The survey specifically noted that freshwater quality improvement measures could include such things as establishing riparian buffers, water management and improvements in wastewater treatment. Survey questions were explicitly worded to remind respondents that a variety of ecosystem services may be affected by species recovery investments. Consequently, the values derived in this survey potentially aggregate a broad selection of ecosystem services likely to accrue to residents from substantial increases in water quality and the rehabilitation of coastal wetlands.

Respondents’ WTP were compared within and across surveys to assess the relative magnitude and credibility of non-use values for little-known aquatic species, thus increasing their utility for inclusion in future benefits transfer analyses. To maintain clarity, here we report only core multinomial logit regression results, without demographic, attitudinal, or survey-specific covariates.

## Case Study

### Location

Ontario is a large (>917,000 km^2^ land, >158,000 km^2^ water area) province in central Canada. In 2006, it had a population of 11.98 m residents living in 4.56 m households (http://www40.statcan.ca/l01/pro01/pro106-eng.htm). Aquatic ecosystem stress levels are high in southern Ontario due to the combination of land cover change, urban and agricultural development, wetland loss, siltation, water quality degradation, over-exploitation of aquatic species, and the presence of alien invasive species (Statistics Canada 2006). The species used in the surveys were largely resident only in southern Ontario, although lake sturgeon is distributed widely within and outside the province (but at very low levels relative to historic abundance) (DFO 2008).

### Canada’s Species at Risk Act (SARA)

The *Species at Risk Act* (SARA) arose from Canada’s obligations under the 1992 United Nations Convention on Biological Diversity (Species at Risk Act 2002) and was enacted in 2003 (Environment Canada 2003). Its purposes are to prevent wildlife species from being extirpated or becoming extinct, to provide for the recovery of wildlife species that are extirpated, endangered, or threatened as a result of human activity, and to manage species of special concern to prevent them from becoming endangered or threatened. The Committee on the Status of Endangered Wildlife in Canada (COSEWIC) assesses Canadian wildlife species (www.cosewic.gc.ca) and makes recommendations for official listing to the Government of Canada but COSEWIC’s recommendations impose no responsibility on the government to list a wildlife species (Mooers et al. 2010). Science advice plays an important role in the process (DFO 2005). A number of authors provide recent perspectives on the performance of the SARA (Mooers et al. 2007; Findlay et al. 2009; Hutchings and Festa-Bianchet 2009; Mooers et al. 2010).

While cost-benefit analysis (CBA) is not a requirement under SARA, listing a species is considered a regulatory change and is thus subject to government CBA guidelines (Treasury Board Secretariat 2007). One objective of this research was to quantify non-use values for little-known species so that the benefits of species protection and recovery could be economically accounted for in decisions about listing such species.

## Methods

### Choice Experiments

Choice experiments (CEs) compare products or policies—SARA recovery scenario outcomes in this survey—that are composed of distinct attributes. Each attribute contributes value to the overall product ‘bundle’ and can be described by the discrete levels it takes on. CEs ask survey respondents to choose their preferred option from among at least two scenarios. When a monetary cost or fee is included as an attribute in CEs, it is possible to derive compensating surplus (willingness-to-pay (WTP)) for changes in non-market ecosystem service attributes.

Assume that Ontario households face *M* discrete alternatives (*A*_1_, *A*_2_, …, *A*_*m*_) relating to SARA conservation programs, each of which provides a certain level of utility, *u*, and that the utility derived from each alternative *A*_*m*_ is denoted as *u*_*m*_. Also assume that utility for each conservation alternative is known with certainty by the household decision-maker. Household *i* will choose conservation program alternative *m* if and only if *u*_*im*_ > *u*_*im*_*’* for every *A*_*m*_ where *m* ≠ *m’*. Because there are aspects of true utility that the researcher cannot observe, an indirect utility function is estimated (see Train 2003) as *u*_*im*_ = *v*_*im*_ + *ε*_*im*_, where *v*_*im*_ is the observed component of utility and *ε*_*im*_ captures factors that affect utility but are unobserved by the researcher.

As the researcher does not have any knowledge about the unobserved portion of utility for each household, they are treated as random. The probability that decision maker *i* chooses alternative *m* is thus *P*_*im*_ = Prob (*ε*_*im*_*’* − *ε*_*im*_ < *v*_*im*_ − *v*_*im*_*’*) for every *m* ≠ *m’*. Under the further assumption that the error term takes on a Gumbel distribution, the theoretical model of utility maximizing behavior can be estimated empirically with a multinomial logit model (Train 2003). Utility is commonly specified to be linear in parameters, *V*_*im*_ = *β’x*_*im*_ where *x*_*im*_ is a vector of observed variables relating to alternative *m* so that the resulting probability of household *i* choosing option *m* is

1 $$\mathop P\nolimits_{im} = \frac{{\mathop e\nolimits^{{\beta 'x_{im} }} }}{{\sum\nolimits_{M} {\mathop e\nolimits^{{\beta 'x_{im} }} } }}$$

Using regression coefficients, marginal WTP can then be calculated for changes in any attribute *i* by the ratio −*β*_*i*_*/β*_fee_, where *β*_fee_ is the coefficient for the cost variable.

### Survey Instrument

Draft surveys were developed for DFO in 2009 and underwent refinement over two years based on internal review, focus group testing, and a pilot survey. Each of the three choice experiments presented each of that survey’s respondents with eight choice tasks that asked them to identify their preferred recovery program scenarios. In each survey, recovery Options A and B varied in their attributes for each choice task, while Option C was always a status quo option. In each of the surveys, the status quo option assumed further declines in listing status for the little-known riverine and coastal wetland species under consideration (e.g., Veisten et al. 2004; Stanley 2005; Lew et al. 2010). Recovery status projections were based on COSEWIC assessments and DFO technical reports. SARA listing changes were embedded within the guild and ecosystem attributes. In all surveys, costs were described in terms of an annual increase in taxes for the next 20 years and assumed to be used entirely for species recovery. Examples of each of the full surveys are included in supporting information S1 to S3.

### Aquatic Species in the Survey

At the time this research was initiated, all species used in the surveys were proposed for listing at various levels under SARA. The three main focal species for this survey were the channel darter, pugnose shiner, and lake sturgeon. The channel darter is a small benthic species of the perch family. Although uncommon in Canada, isolated populations can be found in Ontario and Quebec (COSEWIC 2002a); few survey respondents would be familiar with channel darter. Channel darters generally live in undisturbed rivers along forested or agricultural areas with natural shorelines and good water quality. Freshwater quality improvement measures (e.g., improved farming practices, wastewater treatment, riparian habitat restoration) would play a particularly important role for channel darter recovery. The channel darter was designated as Threatened by COSEWIC.

The pugnose shiner is a timid and secretive small, silvery minnow that seeks cover in clear waters among aquatic plants (COSEWIC 2002b). It has a limited, fragmented Canadian distribution. Declines of the pugnose shiner have been attributed to its extreme sensitivity to decreases in water clarity, loss of habitat from shore development, and destruction of native nearshore aquatic vegetation. Coastal wetland rehabilitation (e.g., wetland purchase, preservation, and rehabilitation) would play a particularly important role for pugnose shiner recovery. The pugnose shiner was designated as Endangered by COSEWIC. Like the channel darter, few respondents would be aware of pugnose shiner.

Lake sturgeon, one of five sturgeon species found in Canada, can weigh up to 180 kg and live over 100 years (COSEWIC 2006). A total of eight Designatable Units (DUs) have been defined in Canada based on freshwater ecological areas, lake sturgeon genetics, and probable historic separation. Historically, commercial fishing caused extreme (99 %) declines in many lake sturgeon populations. More recently, the direct and indirect effects of dams pose important threats. Dams result in habitat loss and fragmentation, altering flow regimes, and may increase mortality by entrainment in turbines. Other threats may include habitat degradation, contaminants, commercial fishing, poaching, and the introduction of non-native species. The lake sturgeon population in DU8 was proposed for listing as Threatened by COSEWIC. Some respondents have likely heard of lake sturgeon and may be aware of the threats that they face in the wild.

In the guild survey, a variety of other riverine and freshwater coastal wetland species were used in conjunction with the channel darter and pugnose shiner. Riverine (channel darter, eastern sand darter, and spotted sucker) and coastal freshwater wetland (pugnose shiner, lake chubsucker, spotted gar, pugnose minnow, warmouth) species tend to have similar habitat requirements and face similar threats within guilds. The riverine species face water quality threats arising from urbanization and agricultural runoff, and the loss of riparian habitat. The coastal wetland species face threats arising from habitat loss due to development and marsh degradation. The ecosystem survey provided examples of riverine and freshwater coastal wetland species that could be expected to benefit from the water quality improvements and increases in coastal wetlands under consideration.

### Species Survey

Four attributes were used for the species survey: (1) SARA listing status of the riverine channel darter; (2) SARA listing status of the coastal wetland pugnose shiner; (3) recovery time of the lake sturgeon; and (4) annual cost (Cdn $1.00 = US $1.01 at the time of the survey) over 20 years (annual income tax increases of $5, $10, $15, $25, $50, or $100 household^−1^ year^−1^). For channel darter, which was proposed for listing as Threatened, the status quo was for listing status to degrade one level further to Endangered. ‘Some improvement’ in recovery trend would keep channel darter at the Threatened level, while a ‘large improvement’ would result in an improvement in listing status to Special Concern. For pugnose shiner, which was being considered for listing as Endangered, the status quo resulted in extirpation, ‘some improvement’ maintained its Endangered status, and ‘large improvement’ improved its listing status to Threatened.

For lake sturgeon within DU8, biological modeling suggested that recovery without any intervention or investment is possible over 170 to 300 years. Five hypothetical recovery strategies (DFO 2008) involved progressively escalating investments to achieve recovery in shorter periods: (1) total closure of the remaining commercial fisheries to increase early adult survival, cutting recovery time to 50–95 years; (2) additionally, increasing minimum size limits to increase late juvenile survival by 10 %, cutting recovery time to 36–67 years; (3) additionally, increasing early juvenile survival by 20 % through habitat rehabilitation and restocking, cutting recovery time to 24–44 years; (4) additionally, maximizing survival of mature adults, cutting recovery time to 19–33 years; and (5) additionally, increasing fertility by 20 % by removing dams, thereby increasing spawning habitat and cutting recovery time to 18–33 years.

### Guild Survey

The three attributes in the guild survey included: (1) SARA listing status of riverine guild species (channel darter, eastern sand darter, and spotted sucker); (2) SARA listing status of coastal wetlands species (pugnose shiner, lake chubsucker, spotted gar, pugnose minnow, warmouth); and (3) annual program cost ($5, $10, $15, $25, $50, or $100 household^−1^ year^−1^) over 20 years.

Table 1 summarizes their projected listing status under different levels of recovery. Note that for the riverine guild there was a net increase of three increments of improvement in listing status at the ‘somewhat improved’ level (i.e., each species is one level higher relative to status quo) and five increments of improvement at the ‘much improved’ level (i.e., channel darter and spotted sucker improve one additional increment but eastern sand darter status remains at Special Concern). For the coastal guild species, there was a net increase of four listing increments for ‘some improvement’ (lake chubsucker remains at Threatened, all others improve by one level) and seven listing increments for ‘large improvement.’ While possible spin-off benefits for other ecosystem services were not mentioned specifically, the nature of potential recovery interventions given as examples likely implied additional benefits to most survey respondents.

**Table 1:** Summary of attributes and levels for the guild survey

	Recommend listing status	Status quo scenario	Some improvement	Large improvement
Riverine species				
Channel darter * Percina copelandi*	Threatened	Degrades to endangered	Remains at threatened	Improves to special concern
Eastern sand darter * Ammocrypta pellucida*	Threatened	Remains at threatened	Improves to special concern	Improves to special concern
Spotted sucker *Minytrema melanops*	Special concern	Degrades to threatened	Remains at special concern	Improves to no longer at risk
Coastal wetland species				
Pugnose shiner * Notropis anogenus*	Endangered	Degrades to extirpated	Remains at endangered	Improves to threatened
Lake chubsucker *Erimyzon sucetta*	Threatened	Remains at threatened	Remains at threatened	Improves to special concern
Spotted gar *Lepisosteus oculatus*	Threatened	Remains at threatened	Improves to special concern	Improves to special concern
Pugnose minnow *Opsopoeodus emiliae*	Special concern	Degrades to threatened	Remains at special concern	Improves to no longer at risk
Warmouth *Lepomis gulosus*	Special concern	Remains at special concern	Improves to no longer at risk	Improves to no longer at risk

### Ecosystem Survey

The three attributes in the ecosystem survey included (1) status of the Water Quality Index (WQI) in southern Ontario; (2) area (ha) of wetlands in the mixedwood plains ecozone (where coastal wetlands occur); and (3) program cost ($5, $10, $15, $25, $50, or $100 household^−1^ year^−1^) over 20 years. Currently, the freshwater WQI is rated as “good” or “excellent” at 60 % of southern Ontario sites, “fair” at 30 %, and “marginal” or “poor” at 10 % (Statistics Canada 2006). ‘Some improvement’ in WQI would improve the mix to 70 % for good or excellent, 24 % for fair, and 6 % for marginal or poor. For a ‘large improvement,’ the mix would further improve to 78 % for good or excellent, 18 % for fair, and 4 % for marginal or poor. The status quo scenario resulted in at least two non-specified species further degrading by one increment and none improving. Under ‘some improvement,’ at least two species improved by one listing increment and none declined in listing status. Under a ‘large improvement,’ at least four species improved by one listing increment or two species improved by two listing increments from the status quo, while none declined in listing status. Thus, relative to the status quo, there was a net increase of at least four listing increments for ‘some improvement’ and six listing increments for a ‘large improvement.’

For the coastal wetland attribute, the status quo was 529,000 ha of wetlands in the mixedwood plains ecozone. This comprises 6.5 % of the total area of this ecozone and is a significant degradation in wetland area from pre-European settlement, when 25 % of the ecozone was comprised of coastal wetlands. A 43,000 ha increase in wetlands from the status quo, ‘some improvement,’ brought total area of wetlands to 572,000 ha. A ‘large improvement,’ where wetlands increased by 125,000 ha, brought total coastal wetlands to 654,000 ha. Under the status quo, at least two species degraded by one listing increment and none improved. Under ‘some improvement,’ at least two species improved by one listing increment and none declined. Under ‘large improvement,’ at least four species improved by one listing increment or two species by two listing increments, and none declined. Again there was a net increase of at least four listing increments for ‘some improvement’ and six listing increments for a ‘large improvement.’ Respondents were also specifically informed that improvements in WQI and coastal wetlands would also have other broad ecosystem impacts beyond species at risk recovery.

### Experimental Design and Survey Testing

The initial survey design was tested with focus group sessions in southern Ontario. Respondent feedback was used to determine the optimal number of choice questions per respondent to maximize information while minimizing respondent fatigue. Each survey was then piloted with a small sample (*n* ≈ 50) from the Ipsos Reid (www.ipsos.ca/en) Ontario internet panel. The fractional factorial experimental design used in the pilot test was then modified by a statistics consultant to formulate efficient experimental designs with Bayesian priors (Sandor and Wedel 2003; Ferrini and Scarpa 2007) from actual survey responses. To reduce potential hypothetical bias, cheap talk script was included in all surveys to remind respondents that species recovery program cost would take away disposable income that respondents could use for other purchases; surveys were clearly identified with Government of Canada logos; respondents were informed that the purpose of the survey was to help government decision-makers better understand citizens’ priorities; and a credible payment mechanism—a tax increase—was used.

The final experimental design for the species survey consisted of five blocks, each of eight choice questions. This survey contained three attributes (the SARA listing status for each of three individual species) with three levels (status quo, some improvement, large improvement) and one cost attribute with six levels. The ecosystem and guild surveys each consisted of three blocks of eight choice questions. These surveys contained two attributes (the collective SARA listing statuses of a group of riverine species, and of a group of coastal wetlands species) with three levels each (status quo, some improvement, large improvement) and one attribute (cost) with six levels. The number of levels and the range for the cost attribute was determined based on focus group testing and pre-test data analysis.

### Sample

Ipsos Reid was contracted by DFO to draw non-random, demographically representative samples from their proprietary Ontario internet panel. Five geographical regions (Greater Toronto Area, Southwest Ontario, Central Ontario, Eastern Ontario, and Northern Ontario) were used to stratify the samples. Regional target quotas were based on a disproportionate sampling plan. Self-reported protestors, respondents who chose the status quo for every choice task and later indicated that they had done so for protest reasons (any one of ‘I do not feel it is my responsibility to pay to protect a species at risk,’ ‘I don’t want more tax added on to what I currently pay,’ or ‘I do not trust the government to effectively run the program’) were flagged in the dataset with a dummy code.

### Data Analysis

An initial latent class (LC) analysis (Vermunt and Magidson 2003) was conducted to identify random and protest respondents. Regression coefficients for random responders were virtually all insignificant while protestors had a highly significant coefficient for the self-reported protest dummy variable and significantly faster than average completion times. Both categories of respondents were eliminated from the dataset.

Effects coding was used for all analyses and the order of non-cost attributes was restricted to be increasing. For the 3-level attributes, this meant that coefficients were centered around ‘some improvement.’ When respondents viewed changes between the status quo and ‘some improvement,’ and between ‘some improvement’ and ‘large improvement,’ as symmetrical the coefficient for ‘some improvement’ was near zero and insignificant. All choice experiment models were estimated with the software Latent Gold Choice (Vermunt and Magidson 2005). Confidence intervals for mean WTP estimates were calculated using the Fieller method (Hole 2007).

## Results

The surveys were conducted online by Ipsos Reid between February 23 and March 2, 2011. A total of 428, 301, and 301 valid completed surveys were collected for the species, guild, and ecosystem surveys, respectively. Ipsos Reid developed weightings for sample respondents by age and gender within regions, then by income, so as to match the 2006 Census profile for residents of Ontario aged 18 or older. Those weights were used in all regression analyses.

### Species Survey

64 protestors (15.0 % of sample) were identified in the initial LC analysis, leaving 364 respondents for the final multinomial logit analysis. The pseudo-*R*^2^ for the model was 0.177, indicating an acceptable fit. All regression coefficients (Table 2) were of expected sign and each species showed significant negative and positive regression coefficients for status quo and ‘large improvement,’ respectively, and insignificant coefficients for ‘some improvement.’ Note that the coefficients for lake sturgeon improvement levels 4 and 5 were the same. With an order restriction in place, this can be interpreted as respondents being willing to pay for improvement up to the fourth level, but not the fifth (recall that lake sturgeon models suggested that additional recovery efforts for level 5—removal of dams to increase spawning habitat—would only reduce recovery time by one year).

**Table 2:** Regression coefficients and WTP (2011 Canadian dollars household^−1^ year^−1^) results

	Model coefficients (*n* = 364; 2912 observations)	Mean WTP ($) and 95 % confidence interval
Darter—status quo	−0.171*	−9.45 (−16.25 to −3.34)
Darter—some improvement	−0.031	–
Darter—large improvement	0.201*	11.14 (4.70 to 18.22)
Shiner—status quo	−0.220*	−12.16 (−19.79 to −5.09)
Shiner—some improvement	−0.014	–
Shiner—large improvement	0.234*	12.95 (5.01 to 22.46)
Sturgeon—status quo	−0.870*	−48.19 (−65.02 to −36.09)
Sturgeon—improvement level 1	0.068	–
Sturgeon—improvement level 2	0.068	–
Sturgeon—improvement level 3	0.133	–
Sturgeon—improvement level 4	0.301*	16.64 (10.89 to 24.22)
Sturgeon—improvement level 5	0.301*	16.64 (10.89 to 24.22)
Cost	−0.018*	
None (opt-out)	−1.822*	−100.91 (−136.77 to −76.88)

### Guild Survey

41 protestors (13.6 % of sample) were identified in the LC analysis, leaving 260 respondents for the multinomial logit analysis of the guild model. The pseudo-*R*^2^ was 0.230, all regression coefficients were of the expected sign, and each species guild showed significant negative and positive regression coefficients for status quo and ‘large improvement,’ respectively (Table 3). Unlike the species model, coefficients for ‘some improvement’ were positive and significant for both riverine and coastal wetland guilds.

**Table 3:** Regression coefficients and WTP (2011 Canadian dollars household^−1^ year^−1^) results for the guild survey

	Model coefficients (*n* = 260; 2080 observations)	Mean WTP ($) and 95 % confidence interval
Riverine guild—status quo	−0.747*	−47.24 (−66.27 to −32.95)
Riverine guild—some improvement	0.269*	16.99 (10.61 to 25.30)
Riverine guild—large improvement	0.479*	30.26 (25.58 to 37.73)
Coastal guild—status quo	−0.758*	−47.93 (−71.10 to −31.77)
Coastal guild—some improvement	0.251*	15.87 (9.79 to 24.05)
Coastal guild—large improvement	0.507*	32.05 (18.58 to 50.45)
Cost	−0.016*	
None (opt-out)	−2.014*	−127.31 (−166.81 to −101.49)

* Significance at the 1 % level

### Ecosystem Survey

30 protestors (10.0 % of sample) and 9 random responders (3.0 % of sample) were identified in the initial LC analysis. One additional respondent with incomplete information was dropped, leaving 261 respondents for the final multinomial logit analysis of the ecosystem model (Table 4). The pseudo-*R*^2^ was 0.222. All regression coefficients were of expected sign and each species showed significant negative and positive regression coefficients for status quo and ‘large improvement.’ Similarly to the guild model, coefficients for ‘some improvement’ were positive and significant for water quality and coastal wetland improvements.

**Table 4:** Regression coefficients and WTP (2011 Canadian dollars household^−1^ year^−1^) results for the ecosystem survey

	Model coefficients (*n* = 261; 2088 observations)	Mean WTP ($) and 95 % confidence interval
WQI—status quo	−1.028*	−62.98 (−83.30 to −48.43)
WQI—some improvement	0.348*	21.29 (14.17 to 29.58)
WQI—large improvement	0.681*	41.70 (34.90 to 53.09)
Wetlands—status quo	−0.572*	−35.05 (−49.68 to −24.62)
Wetlands—some improvement	0.190*	11.61 (4.52 to 20.67)
Wetlands—large improvement	0.383*	23.44 (13.31 to 35.79)
Cost	−0.016*	
None (opt-out)	−1.794*	−109.87 (−146.57 to −84.13)

* Significance at the 1 % level

### Survey Results: Mean WTP

**Fig. 1:**
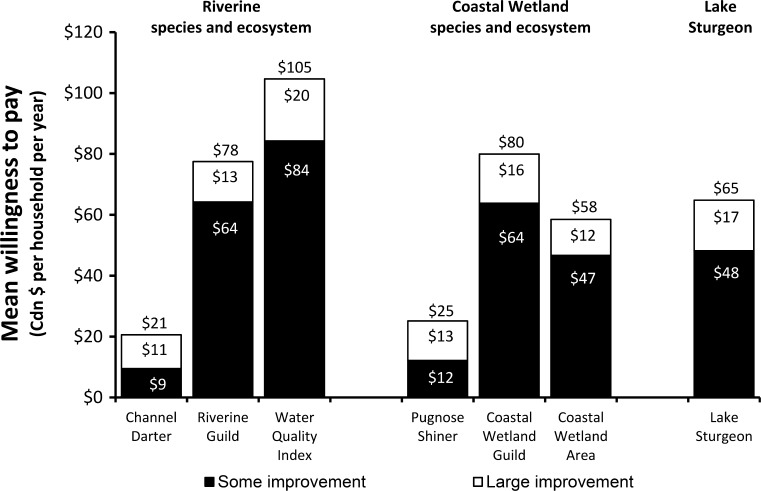
Mean WTP (2011 Canadian dollars) for riverine and coastal wetland species and habitats

Mean WTP for improvements from status quo to ‘some improvement’ levels and from ‘some improvement’ to ‘large improvement’ levels for all attributes from the three surveys are shown in Fig. 1.

The values for individual little-known riverine (channel darter) and coastal wetland (pugnose shiner) species were significantly less than values for guild-based (riverine species: channel darter, eastern sand darter, and spotted sucker; coastal wetland species: pugnose shiner, lake chubsucker, spotted gar, pugnose minnow, warmouth) or ecosystem-based (water quality index; coastal wetland area) attributes. Respondents’ mean WTP for channel darter alone was $20.59 household^−1^ year^−1^ while for three little-known riverine species it was $77.50 household^−1^ year^−1^. Similarly for pugnose shiner, mean WTP was $25.11 household^−1^ year^−1^ for the single species and $79.98 household^−1^ year^−1^ for five little-known coastal wetlands species. Respondents’ mean WTP for reduced lake sturgeon recovery time was as high ($64.83 household^−1^ year^−1^) as for large coastal wetland improvements ($58.49 household^−1^ year^−1^).

WTP per increment in listing status is shown in Table 5. For example, a one-level increase in channel darter listing status relative to the status quo is worth, on average, $9.45 increment^−1^ household^−1^ year^−1^ while a two-level increase is worth $10.30 increment^−1^ household^−1^ year^−1^ (=$20.59/2). For riverine guilds, when there were net increases of three and five SARA listing increments for ‘some improvement’ and ‘large improvement,’ annual household WTP was $21.41 (=$64.23/3) and $15.50 (=$77.50/5) increment^−1^ household^−1^ year^−1^, respectively. For the ecosystem survey, WTP per listing increment was calculated with the total number of generic increments for each attribute and level of improvement.

**Table 5:** Summary of WTP for improvements in SARA listing status (2011 Canadian $ increment^−1^ household^−1^ year^−1^)

	Species model	Guild model	Ecosystem model
Lake sturgeon			
Mean WTP: status quo to large improvement	64.83		
Total number of species	1	–	–
Includes more than non-use values?	Yes	–	–
‘*Some improvement*’			
Mean decrease in recovery time (170–300 to 50–95 years)	152 years	–	–
Mean WTP	$48.19	–	–
Mean WTP per year decrease	$0.317	–	–
‘*Large improvement*’			
Mean decrease in recovery time (170–300 to 19–33 years)	209 years	–	–
Mean WTP	$64.83	–	–
Mean WTP per year decrease	$0.310	–	–
Riverine species			
Mean WTP: status quo to some improvement	9.45	64.23	84.27
Mean WTP: status quo to large improvement	20.59	77.50	104.68
Species	Channel darter	Channel darter Eastern sand darter Spotted sucker	Non-specified (4 for some improvement, 6 for large improvement)
Total number of species	1	3	4/6
Includes more than non-use values?	No	Likely	Yes
‘*Some improvement*’			
Increments in listing status	1	3	At least 4
Mean WTP per species	9.45	21.41	21.07
Mean WTP per listing increment	9.45	21.41	21.07
‘*Large improvement*’			
Increments in listing status	2	5	At least 6
Mean WTP per species	20.59	25.83	17.45
Mean WTP per listing increment	10.30	15.50	17.45
Coastal wetland species			
Mean WTP: status quo to some improvement	12.16	63.80	46.66
Mean WTP: status quo to large improvement	25.11	79.98	58.49
Species	Pugnose shiner	Pugnose shiner Lake chubsucker Spotted gar Pugnose minnow Warmouth	Non-specified (4 for some improvement, 6 for large improvement)
Total number of species	1	5	4/6
Includes more than non-use values?	No	Likely	Yes
‘*Some improvement*’			
Increments in listing status	1	4	At least 4
Mean WTP per species	12.16	12.76	11.67
Mean WTP per listing increment	12.16	15.95	11.67
‘*Large improvement*’			
Increments in listing status	2	7	At least 6
Mean WTP per species	25.11	16.00	9.75
Mean WTP per listing increment	12.56	11.43	9.75

Ontario’s population lives in 4.56 million households (2006 Census). Conservatively assuming that the 15.0 % protestors had zero WTP for aquatic species recovery, the species survey implies non-use values of $79.8 million year^−1^ (=$39.9 million increment^−1^ year^−1^) in Ontario for a 2-level difference in channel darter listing status (degrading to Endangered versus improving to Special Concern). For a two-level improvement in pugnose shiner listing status (degrading to Extirpated versus improving to Threatened), non-use values would be $97.3 million year^−1^ (=$48.7 million increment^−1^ year^−1^). Using a 7 % discount rate over the 20 year time period, this equates to a present value of $845 million and $1031 million for two-level improvements in the listing status for channel darter and pugnose shiner, respectively. Policy-makers would be able to justify the costs of conservation programs up to these levels on economic grounds, provided the programs were expected to achieve the associated improvements for the species.

For riverine and wetland guilds and assuming 13.6 % protestors, non-use values for large improvements were $305.3 million year^−1^ and $315.1 million year^−1^, respectively. This translates to $101.8 million species^−1^ year^−1^ and $61.1 million increment^−1^ year^−1^ for riverine species, and $63.0 million species^−1^ year^−1^ and $45.0 million increment^−1^ year^−1^ for coastal wetland species. At the broadest scope, assuming 13.0 % protestors and random responders, survey results implied total WTP for large improvements in water quality and coastal wetland area in southern Ontario were $415.3 million year^−1^ and $232.0 million year^−1^, respectively. Based on an increase of 125,000 ha in the large improvement scenario, this equates to $1850 ha^−1^ year^−1^. For the unspecified aquatic species that would recover as a result of these improvements in environmental conditions, this translates to $69.2 million increment^−1^ year^−1^ and $103.8 million species^−1^ year^−1^ for riverine species, and $38.7 million increment^−1^ year^−1^ and $58.0 million species^−1^ year^−1^ for coastal wetland species.

Lake sturgeon values were calculated on reductions in recovery time rather than improvements in listing status. Assuming 15.0 % protestors, the aggregate value for improving from status quo (170–300 years until recovery) to the first level improvement (50–95 years until recovery) was $186.8 million year^−1^. The next significant level for WTP was at improvement level 4, where recovery time fell to between 19 and 33 years; mean WTP increased to $64.83 household^−1^ year^−1^, an aggregate value of $251.3 million year^−1^. WTP for reductions in lake sturgeon recovery time was nearly constant at $0.31 household^−1^ year^−1^, which translates to an annual value of $1.41 million year^−1^ for each year lake sturgeon recovery was accelerated.

## Discussion

Clear, positive, and significant non-use values were found for little-known aquatic species at risk in southern Ontario. The WTP estimates seem broadly in accordance with results from prior individual studies and meta-analyses. For example, in a meta-analysis of 60 contingent valuation studies of threatened and endangered species, Martín-López et al. (2008) found mean WTP of $75 and $34 household^−1^ year^−1^ for securing gains and avoiding losses in biodiversity, respectively. Johnston et al. (2005) summarized values from 34 studies focusing specifically on aquatic species, finding that WTP ranged from approximately $25 to $750 household^−1^ year^−1^ for water quality changes that affected aquatic life habitats and/or recreational fishing and other recreational uses. The valuation results appear reasonably relative to results from past research on aquatic species at risk. Many factors can, however, influence WTP and need to be considered.

### Potential Biases

It is well known that a variety of embedding or ‘adding up’ issues can arise when valuing biological diversity at different levels of aggregation (e.g., Lew and Wallmo 2011; Christie et al. 2006). In an extreme example, Jacobsen et al. (2007) found that WTP for one named little-known moth, *Euxoa lidia*, was worth as much ($74.13 household^−1^ year^−1^) as 25 unnamed species resident in heath habitats ($74.63 household^−1^ year^−1^). Using CEs, rather than contingent valuation, can ameliorate embedding problems (Adamowicz et al. 1998; Hanley et al. 2001). Lew and Wallmo (2011) conducted 46 external scope tests for valuation estimates derived by choice experiments for three aquatic species at risk. They found some scope insensitivity but also that scope effects were proportional to the number of species in most cases and concluded that their results supported well-behaved preferences for threatened and endangered species in their study’s context.

The ‘iconization’ effect can also have an important effect on results when specifically naming species in a survey; naming species can ‘break anonymity’ (Jacobsen et al. 2007). Social desirability biases can exert substantial influence in surveys that use hypothetical choices (Lusk and Norwood 2009a, b; Norwood and Lusk 2011). Norwood and Lusk (2011) suggested that socially desirable behaviors might lead to a greater likelihood of respondents’ indicating a preference for selecting one of the choice tasks compared to the status quo or indicating stronger preferences for goods with ‘normative’ attributes. Implicit social pressure to make such choices often exists because the benefits of that choice extend to society as a whole.

To address other sources of hypothetical bias, the survey design used approaches which have been found to reduce bias (Loomis 2013): the surveys emphasized consequentiality of the respondents’ choices, urged honesty in the responses, and included a cheap talk script.

### Credibility of WTP Estimates

Given the numerous factors that can positively or negatively affect WTP estimates, how reasonable are the WTP estimates for little-known species from this research? Mean WTP for the two little-known species are statistically similar (Table 2). Mean WTP for lake sturgeon, a better known species that likely provides additional benefits beyond non-use values, was about 2.5 times higher. This is assuring because little- and well-known species values seem plausible when compared to each other.

There was sensitivity to scope among riverine species as WTP for one species was less than WTP for multiple species (recall Table 5). For instance, WTP increased from $9.45 and $20.59 to $21.41 and $25.83 per species for ‘some’ and ‘large’ improvements, respectively. Increasing returns (i.e., higher values per fish or listing increment) to additional riverine species conservation efforts suggest that WTP for the guild-based survey may be capturing broader ecosystem service benefits and are not restricted only to non-use values.

When comparing WTP for coastal wetland species, the trends were different. Mean WTP for ‘some improvement’ was $12.16 household^−1^ year^−1^ for only the pugnose shiner and $12.76 species^−1^ household^−1^ year^−1^ for a suite of five species that included the pugnose shiner. For ‘large improvement,’ mean WTP was $25.11 household^−1^ year^−1^ for the pugnose shiner alone but only $16.00 species^−1^ household^−1^ year^−1^ for the 5-species guild. While the mean WTP per riverine guild species increased 25 % ($20.59 to $25.83) relative to channel darter alone, mean WTP per wetland guild species declined 32 % ($25.11 to $16.00). Sampling procedures and survey formats were similar across surveys, so the difference more likely arose due to differences in broad ecosystem service benefits provided from improved WQI versus rehabilitated coastal wetland area.

Freshwater provides a broad range of ecosystem services, and water quality is a very important issue in Canada (Rudd et al. 2011). This is particularly so for respondents in southern Ontario, where in May 2000 there was widespread illness and several deaths from drinking contaminated water in the town of Walkerton (Hrudey et al. 2003). That event highlighted the importance of water quality for human health and prompted major reviews and revisions in standards for controlling water pollution in Ontario. While coastal wetlands also provide a broad range of ecological functions and ecosystem services (Brander et al. 2006), the notable difference between WQI and wetlands may be their human health effects and help explain the differences in WTP. Overall, we believe the survey results for species- and guild-based surveys are coherent and reasonable when compared simultaneously.

In the riverine-oriented surveys, per species WTP remained constant for the intermediate level of improvement between the guild and ecosystem surveys ($21.07 versus $21.41 species^−1^ household^−1^ year^−1^) but declined with a large improvement ($17.45 versus $25.83). For the wetland surveys, mean WTP per species declined from $12.76 to $11.67 for ‘some improvement’ and from $16.00 to $9.75 for ‘large improvement.’ The results suggest that mean WTP for large improvements in the status of five little-known species in the coastal wetland guild survey ($79.98) was higher than for increasing coastal wetland area by 125,000 ha ($58.49), a measure that would benefit these five species plus provide additional ecosystem service benefits. This seems implausible. The ecosystem survey reminded respondents that if “WQI improves, populations of aquatic species at risk may stabilize or increase in their native areas [and that] other freshwater fish that are not at risk would also benefit from WQI improvement, as would a variety of birds, molluscs, plants, and terrestrial animals.” For coastal wetlands, the survey reminded respondents that wetlands “also provide other benefits to humans, including: filtration of water; flood retention; erosion reduction; recreation opportunities (canoeing, fishing, bird watching); harvesting (berries, grains); carbon storage; nutrient cycling; and groundwater recharge.”

The discrepancy between mean WTP across the surveys implies that either ecosystem benefits should be higher or that WTP estimates in the species- and guild-based surveys should be lower. There may be valid reasons for believing that mean WTP from the ecosystem-based survey is somewhat low from a purely WQI-oriented perspective. Van Houtven et al. (2007) used a 10-point water quality index based on a water quality ladder (i.e., steps from boatable to fishable to swimmable water quality) for their water valuation meta-analysis (*n* = 21 studies). They found mean WTP of $60, $138, and $233 for 1, 3, and 6 unit changes in water quality for water users ($60, $46, $39 per unit), and $21, $48, and $77 for non-users ($21, $15, $13 per unit). There were diminishing returns in that meta-analysis but it is difficult to translate WTP measures for indices that vary in construction. Hanley et al. (2006) reported mean WTP of $66 to $77 for ‘large improvements’ in river ecology in their UK water quality study. Magat et al. (2000) estimated that mean WTP for a 15 % increase in water quality was $617 in their Colorado study, and that $199 of that total was attributable only to rivers. Our results (mean WTP = $105 household^−1^ year^−1^) for large improvements in water quality may be at the low end of this range but do not appear unreasonable compared to other valuation exercises.

For coastal wetlands ecosystem services, the estimate of mean WTP of $1850 ha^−1^ year^−1^ is within the $5260 mean and $282 median WTP range from Brander et al.’s (2006) wetland meta-analysis. The surveys did not ask respondents to value all ecosystem services but to focus on improvements in WQI and coastal wetlands that would generally help protect aquatic species at risk; it could be that the value was comprised of non-use values plus some portion of other ecosystem service benefits accruing to Ontario residents.

A second possibility is that species and listing status increment values in the species- and guild-based survey are too high relative to WTP values calculated with the ecosystem-oriented survey. Unnamed species were used in the ecosystem survey; iconization of named species in the species- and guild-based surveys may have had a substantial effect on WTP estimates. Jacobsen et al. (2007) found that Danish households’ WTP for one named little-known species was as high as WTP for 25 unnamed species, illustrating that iconization effects can be substantial. While social desirability bias could also lead to unrealistically high WTP values, we would expect social desirability biases to be consistent across our three surveys irrespective of iconization of species within the surveys. We thus suggest that naming species in two surveys and not naming them in the broader ecosystem-based survey is the most likely source of the seeming anomalies in WTP among the surveys. Intuitively it does not seem reasonable that overall WTP should decline in either riverine- or wetland-oriented ecosystem surveys. With survey wording emphasizing broader ecosystem benefits, higher values should be more likely in the ecosystem survey relative to others.

### Potential for Calibration of Economic Value

If there are truly discrepancies in mean WTP between surveys due to iconization, one option may be to calibrate WTP across survey types. When named species values were recalibrated to be 33 % of their current level, the overall ratio of values seem to be much more reasonable. Might it be more reasonable to scale down WTP estimates for little-known species or increase per species benefits from the ecosystem estimates? This issue should be relatively easy to follow up on with specific research testing for WTP differences between WQI and coastal wetland improvements in the presence and absence of named species information. Inferred value surveys could be used to test for biases in WTP from surveys at all levels of ecosystem service aggregation. Such research should allow a more definitive assessment as to what degree ecosystem service values are ‘too low’ and species values ‘too high’ and would help narrow the range of credible WTP values for little-known species important for policy purposes. Note that even with a 50–70 % downward recalibration of current WTP values for little-known species, their aggregate value would still be in the tens of millions of dollars per year.

### Relevance for Freshwater Conservation Policy

One objective of this research was to quantify non-use values for little-known species so that the economic benefits of species protection and recovery could be accounted for in decisions about listing such species under Canada’s SARA. In the context of threatened, endangered, or rare marine species, Lew (2015) highlighted several ways policy-makers and analysts could use such economic values: as inputs in ecosystem-based management models that enable the fuller accounting of the scope and magnitude of the private and social benefits and costs associated with policies affecting aquatic biodiversity and other resources; for formally evaluating in a CBA framework the economic trade-offs between multiple resource uses; and in natural resource damage assessments. Sanchirico et al. (2013) illustrated how, in CBA, including economic values associated with protecting an endangered aquatic species could significantly affect policy recommendations from an economic efficiency perspective.

To account for the benefits of species protection and recovery for little-known freshwater aquatic species in Canada, economic value information for such species—or for species that are sufficiently similar—must be available. In his recent review, Lew (2015) found over 30 published studies from the past few decades that measured economic values associated with the preservation, protection, and enhancement of marine species. However, the field suffers from coverage issues, both in terms of geographical area (primarily developed countries) and species types (primarily charismatic megafauna with only a small handful of lesser known species). The availability and quality of economic values for freshwater species are even more limited (Grantham and Rudd 2015). The overall result is a severe constraint in information that can be used to support policy analysis and decision-making for little-known freshwater species. In Canada, such information is crucial to help inform analyses in support of listing decisions under national legislation to conserve and recover species at risk.

Policy analysts wishing to use such economic value information rarely have the time or resources to conduct primary research to obtain those values. Instead, benefits transfer methodology (e.g., Johnston et al. 2005; Brander et al. 2006; Hanley et al. 2006) is often used, where economic values derived in one particular policy context are then transferred and used in other similar contexts where primary valuation research was not available. The more closely a researcher can customize the value estimate to the new policy application, the more accurate the transferred value will tend to be relative to the value that would be generated if a primary study had been conducted (Lew 2015). Given the values derived from multiple levels and given ecological similarity between various little-known riverine and coastal wetlands freshwater species, it is reasonable to assume that the values derived in this study will be useful for future freshwater fish economic meta-analyses and applicable for benefits transfer in a wide variety of policy contexts in Canada, and potentially beyond. The study also provides support for the idea that groups of species that occupy similar ecological niches in southern Ontario rivers or coastal wetlands could be valued together at the guild level, thus increasing the potential efficiency of valuation research targeting aquatic species at risk.

## Conclusion

This research helped to narrow in on credible economic values for little-known aquatic species at risk in Canada. The results show broad congruence with values calculated in other parts of the world, so we suggest that there can be an increasing level of confidence that valid non-use values for little-known species do, in fact, exist and can be quantified. For little-known aquatic species, we suggest reasonable benefits transfer estimates may be $10 to $25 species^−1^ household^−1^ year^−1^ or $10 to $20 increment^−1^ household^−1^ year^−1^ for improvements in listing status. Even if calibration of the values from non-named species are needed, the valuation estimates for benefits transfer set a baseline which can be further refined in the future. While this still leaves a substantial degree of uncertainty for benefits transfer applications, we anticipate that sensitivity analyses (i.e., Akter and Grafton 2010) within that range of values would prove useful for many environmental management and resource allocation decisions. This research highlighted the value of conducting multiple surveys at different levels of ecosystem service aggregation in parallel. While species-based surveys provide useful information on non-use values, the defensibility of species-based WTP estimates may be substantially enhanced when surveys at higher levels of ecosystem service aggregation are conducted in parallel with species-oriented surveys.

## Electronic supplementary material

Below is the link to the electronic supplementary material.

Supplementary material 1 (PDF 462 kb).S1. Example (pdf) of a full survey for species

Supplementary material 2 (PDF 419 kb).S2. Example (pdf) of a full survey for guilds

Supplementary material 3 (PDF 465 kb).S3. Example (pdf) of a full survey for ecosystems
