# Supplementary material for: Non-use Economic Values for Little-Known Aquatic Species at Risk: Comparing Choice Experiment Results from Surveys Focused on Species, Guilds, and Ecosystems
Source: Environ Manage. 2016 Jun 13;58:476–90. doi: 10.1007/s00267-016-0716-0 (PMC4969348; doi:10.1007/s00267-016-0716-0)
Supplement: Supplementary file 1 — Supplementary material 1 (PDF 462 kb).S1. Example (pdf) of a full survey for species [file 267_2016_716_MOESM1_ESM.pdf]

Supplementary Material S1: Example of a full survey for species

Article title: Non-use economic values for little-known aquatic species at risk: comparing choice experiment results from surveys focused on species, guilds and ecosystems
Journal name: Environmental Management
Authors: Murray A. Rudd[1], Sheri Andres[2]*, Mary Kilfoil[3,4]
[1]Department of Environmental Sciences, Emory University, Atlanta, USA
[2]Policy and Economics – Central and Arctic Region, Fisheries and Oceans Canada, Winnipeg, Manitoba, Canada
[3]Gardner Pinfold Consulting Economists Ltd., Halifax, Nova Scotia, Canada
[4]Present address: Rowe School of Business, Faculty of Management, Dalhousie University, Halifax, Nova Scotia, Canada
* Corresponding author: Sheri.Andres@dfo-mpo.gc.ca

DEPARTMENT OF FISHERIES AND OCEANS
Conservation of Aquatic Species at Risk in Ontario – Species Survey
FINAL: February 8, 2011

Introduction at Site

[INSERT STANDARD PANEL INTRODUCTION]

Screening

S1. What is your age?

Month / Year of birth [NUMERIC FIELD]

[TRACK AGE QUOTAS BASED ON S1]

S2. What is your gender?

Please select one response only

Male
Female

[TRACK GENDER QUOTAS BASED ON S2]

S3. In what country do you live?

Please select one response only

USA
Canada
Australia
United Kingdom

Other

[CONTINUE IF CANADA, ELSE THANK & TERMINATE]

S4. In which of the following provinces do you reside?

Please select one response only

Newfoundland and Labrador
Prince Edward Island
Nova Scotia
New Brunswick
Quebec
Ontario
Manitoba
Saskatchewan
Alberta
British Columbia
Yukon Territory
Northwest Territories
Nunavut

[CONTINUE IF ONTARIO, ELSE THANK & TERMINATE]

S5. ONTARIO REGION [DO NOT ASK – AUTOFILL FROM PANEL INFO]

GTA
Southwest Ontario
Central Ontario
Eastern Ontario
Northern Ontario

[TRACK REGION QUOTAS BASED ON S5]

S6. What was the total income for all members of your household before taxes in 2010?

Please select one response only

Less than $20 000
$20,000 to $39,999
$40,000 to $59,999
$60,000 to $79,999
$80,000 to $99,999
$100,000 to $124,999
$125,000 to $149,999
$150,000 or more

[TRACK INCOME QUOTAS BASED ON S6]

PANEL DEMOGRAPHICS [APPEND THE FOLLOWING PANEL INFORMATION]

Age
Gender

FSA
Ontario Region
CSD (census subdivision) Name
CMA/CA (Census Metropolitan Area/Census Agglomeration)
Household Income
Education
Employment Status
Occupation (primary panellist)
Own or Rent
Household Size
Number of Children in the Household
Marital Status (primary panellist)

[THIS INFORMATION IS REQUIRED FOR: NON-RESPONDERS, DQs/OVER QUOTA, PARTIAL COMPLETES AND COMPLETES]

Conservation of Aquatic Species at Risk in Ontario

Fisheries and Oceans Canada Pêches et Océans Canada
Canada

Welcome!

The purpose of this survey is to help government decision-makers better understand the priorities of citizens with regard to the conservation of aquatic (fish, molluscs, reptiles) species at risk in Ontario.

Your participation in this survey is **voluntary** and you may decide to stop participating in the survey at any time. **The information that you provide is important!** We very much appreciate the time and effort you take to complete this survey.

Your answers to the survey will be kept private. Any reports about this study will not identify you in any way. Results will be shown in group form only. None of the personal identifying information you provided to Ipsos when you joined the i-Say panel will be shared with any other individual, organization, or government agency.

Government Priorities

Government Priorities

1. The Government of Canada spends money in a wide variety of areas that directly or indirectly help improve the quality of lives of Canadians. Personal income taxes provide over 49% of the funds used to pay for these services and activities (click here for more information). **[ENSURE THE HYPERLINK OPENS AS A NEW WINDOW]**

In your opinion, how important is it for the Government of Canada to invest in each of the following factors? Please use a scale of 1 to 5 where **1** means **not at all important** and **5** means **very important**.

Please select one response for each item

[ACROSS TOP OF GRID]
1 – Not at all important
2
3
4
5 – Very important

[DOWN SIDE OF GRID] [RANDOMIZE ORDER]
Maintain and build public infrastructure in Canada
Increase overall economic activity (GDP) in Canada
Reduce poverty and inequalities in wealth within Canada
Participate in efforts to reduce conflict and enhance security in Canada and abroad
Develop an active and vibrant arts and culture sector in Canada
Improve the physical and mental health of Canadians
Increase employment opportunities for Canadians
Increase the number of Canadians graduating from high schools, colleges and universities
Build social cohesion and trust in Canadian society
Protect our environment, ecosystems, and biodiversity

The *Species at Risk Act* (SARA)

A number of aquatic species in Canada are listed as species at risk of extinction. Species at risk are protected under the *Species at Risk Act* (SARA) (2003). SARA protects not only the species themselves but also their habitat. According to this Act, a species becomes more at risk as it passes from **Not At Risk** to **Special Concern** to **Threatened** to **Endangered**.

The figure below defines each of the terms used in SARA.

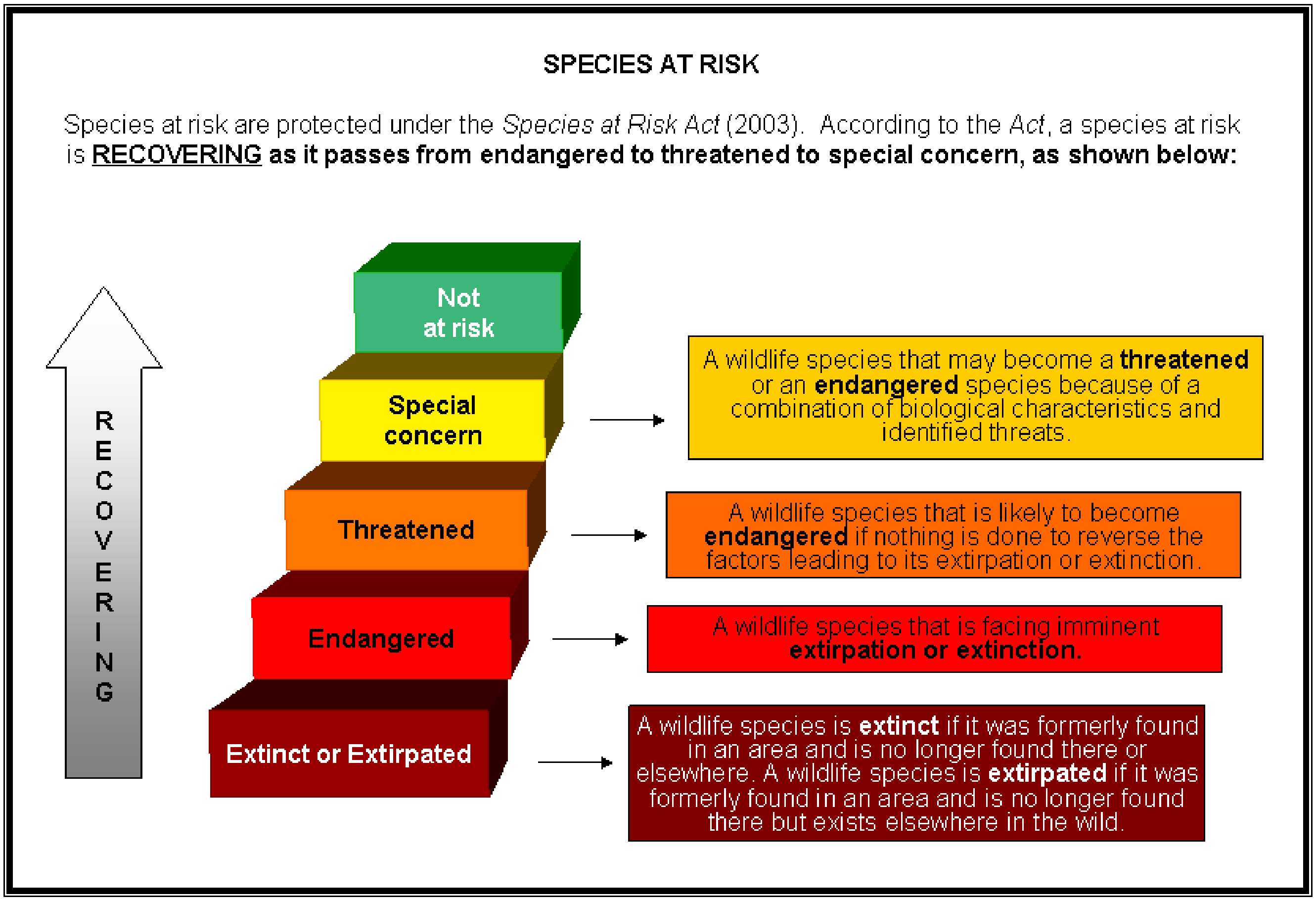

Click here **[INSERT LINK TO 'SARA Ontario POP-UP' – ENSURE IT OPENS AS A NEW WINDOW.]** for a list of species in Ontario that are already protected under SARA or are under consideration for addition to SARA.

2. Before starting this survey, how familiar were you with the ***Species at Risk Act* (SARA)**?

Please select one response only

Very familiar
Somewhat familiar
Not familiar

Threats to Aquatic Species in Ontario

Threats to Aquatic Species in Ontario

3. A number of factors could threaten various aquatic species in Ontario. **In your opinion, how important are each of the following potential threats to aquatic species in Ontario?** Please use a scale of 1 to 5 where **1** means **not at all important** and **5** means **very important**.

Please select one response for each item

[ACROSS TOP OF GRID]
1 – Not at all important
2
3
4
5 – Very important
Don't know

[DOWN SIDE OF GRID] [RANDOMIZE ORDER]
Water pollution
Recreational fishing
Competition from non-native (invasive) species (e.g. zebra mussels)
Acid rain
Aquaculture (fish farming)
Commercial fishing
Climate change
Habitat loss/degradation from agricultural activities
Habitat loss/degradation from urbanization
Habitat loss/degradation from industrial activities (e.g. oil and gas, forestry and mining exploration/extraction)
Habitat loss/degradation, habitat fragmentation and species mortality/injury from dams and other barriers in rivers and streams

Threats to Aquatic Species in Ontario

4. Are there **other factors** that you think pose a threat to aquatic species in Canada?

Please be detailed and specific in your response. You may enter 'No' or 'Don't know.'

[VERBATIM RESPONSE]

Species Recovery Investment Preferences

In this section of the survey, we are going to show you a series of 8 questions in which you **choose your preferred SARA investment option from two hypothetical alternatives, plus the option to not invest**.

The SARA investment options may vary in their ultimate impacts on species abundance (population size) and listing status, on species habitat, and on their cost. The options will be described using the following characteristics:

- impact of the recovery investment on the SARA status of the **Channel Darter**.
- impact of the recovery investment on the SARA status of the **Pugnose Shiner**.
- impact of the recovery investment on the SARA status of the **Lake Sturgeon**.
- annual cost of the program to your household.

In the questions that follow, we will ask you to indicate which of two recovery investment options, if either, **you would be willing to pay for**.

The following pages will present more information about how the recovery investment options vary.

Channel Darter Background Information

The Channel Darter is a small bottom-dwelling fish of the Perch family. This fish is light sand or olive in color with brown speckles on its back. Adults are commonly 34 to 63 mm (1.3 to 2.5 in) in total length, although adults up to 73 mm (2.9 in) have been found.

(insert channel darter image) (center image on screen)

The Channel Darter is **currently listed as Threatened under SARA**.

Although the Channel Darter is uncommon in Canada, isolated populations are found in Ontario and Quebec. In Ontario, adults are found in riffle margins (edges around a shallow area where rocks break up the flow of water) of small to medium-sized rivers, and gravel lakeshore beaches where the current is slow.

High quality freshwater habitat is particularly important for the survival of the Channel Darter in Ontario. Channel Darters are threatened by the loss of suitable habitat because they are sensitive to sedimentation, shoreline modifications and decreased water quality caused by agricultural or urban development.

Populations of Channel Darters may stabilize or increase in their native areas when water quality improves. **Freshwater quality improvement measures**, such as:

- improved farming practices, and
- riparian habitat (vegetated area along the sides of a stream) restoration

would play an important role for Channel Darter recovery.

Pugnose Shiner Background Information

The Pugnose Shiner is a small, silvery minnow. Its mouth is very small and upturned. Maximum total length is 60 mm (2.4 in) for females and 50 mm (2.0 in) for males. The Pugnose Shiner is typically found in clear, heavily-vegetated lakes and embayments, and slow-moving streams.

(insert pugnose shiner image) (center image on screen)

The Pugnose Shiner has been found in four general locations in Ontario. Of the 11 known populations, only 3 are considered "fair" or "good" and at least 3 are extirpated.

The Pugnose Shiner is **currently designated as Endangered under SARA**. It has a limited, fragmented distribution, and in Canada is found only in Ontario, where it is subject to declining habitat quality.

Coastal lakeshore and wetland habitat is extremely important for the Pugnose Shiner. Declines of the Pugnose Shiner have been attributed to its sensitivity to decreases in water clarity, loss of habitat due to shoreline development, and destruction of native aquatic vegetation.

Populations of Pugnose Shiners may stabilize or increase in their native areas when suitable habitat is available. **Coastal wetland rehabilitation**, such as:

- wetland purchase,
- wetland preservation, and
- wetland rehabilitation

would play a particularly important role for Pugnose Shiner recovery.

Lake Sturgeon Background Information

The sturgeon family contains 24 species, 5 of which are found in Canadian waters. One, the Lake Sturgeon, is found only in fresh water and is one of Canada's largest freshwater fishes reaching lengths of up to 3 m (10 feet) and weights of up to 180 kg (400 lbs). They can live over 100 years, and are considered "living fossils" as they are very similar to their ancestors in the Devonian period (some 350 million years ago.

(insert lake sturgeon images) (center images on screen)

A **Designatable Unit (DU)** is a significant and irreplaceable unit of biodiversity – meaning that if this particular group was lost, it likely would **not be replaced naturally**. Eight DUs of Lake Sturgeon have been defined in Canada (map) **[INSERT HYPERLINK TO 'DU Map POP-UP' – ENSURE IT OPENS IN A NEW WINDOW]** based on freshwater ecological areas, Lake Sturgeon genetics, and probable historic separation. DU8 (Great Lakes-Upper St. Lawrence) occurs in southern Ontario and Quebec.

Historically, commercial fishing during the mid-1800s to early 1900s caused drastic (99%) declines in the majority of Lake Sturgeon populations in DU8. Since then, most [illegible] the 63 populations in DU8:

Lake Sturgeon (Photo credit Eric Engbretson/ CritterZone.com)

- 21 populations are now considered extirpated
- only 20 populations are known to spawn successfully
- only 4 of the spawning populations are considered large
- some populations are showing signs of modest recovery

The Lake Sturgeon in DU8 is **currently under consideration for listing under SARA as Threatened**. The full status report is available here. **[ENSURE HYPERLINK OPENS IN A NEW WINDOW]**

Current threats to Lake Sturgeon in DU8 include:

- Direct and indirect effects of dams. Dams result in habitat loss and fragmentation, altering flow regimes, and may increase mortality by entrainment (incidental trapping) in turbines.
- Habitat degradation resulting from inappropriate land use and/or poor agricultural practices
- Other threats may include contaminants, commercial fishing, poaching, and the introduction of non-native species (such as zebra mussels).

Lake Sturgeon Recovery Options

Preliminary recovery targets have been developed for Lake Sturgeon in DU8 (technical report available here). The current population of Lake Sturgeon is likely less than 1% of the historic overall population in the Great Lakes and their surrounding watersheds. Recovery would entail an overall population of approximately 12,000 adult spawners.

Biological modeling suggests that recovery without any intervention or investment is possible over 170 to 300 years (depending on assumptions about current population level and mortality rates).

Various actions could be taken to achieve recovery over a shorter time period with less risk.

Lake Sturgeon Recovery Strategies

Five hypothetical recovery strategies are presented below, along with a no investment/no action option. Each recovery strategy involves one additional action that decreases the recovery time. However, each additional action also significantly increases the cost of recovery.

	Strategy					
**Possible Actions**	**No investment**	**Recovery Strategy 1**	**Recovery Strategy 2**	**Recovery Strategy 3**	**Recovery Strategy 4**	**Recovery Strategy 5**
**No action**	✓					
**Total closure of fishery for early adults to increase early adult survival**		✓	✓	✓	✓	✓
**Increase minimum size limits to increase late juvenile survival by 10%**			✓	✓	✓	✓
**Increase early juvenile survival by 20% through habitat rehabilitation and restocking**				✓	✓	✓
**Maximize survival of late adults**					✓	✓
**Add 20% increase in fertility by removing dams (and increasing spawning habitat)**						✓
**Recovery Time**	170 to 300 years	50 to 95 years	36 to 67 years	22 to 44 years	19 to 33 years	18 to 33 years

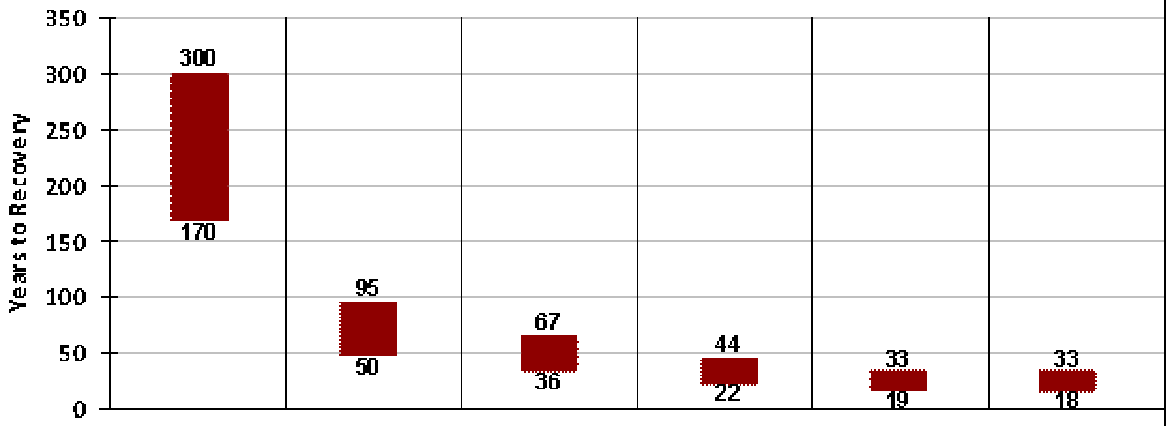

Program Cost

So far, we have described the potential benefits to the three fish species arising from new SARA recovery investments. There is, of course, a need to pay for these investments so the final way that the options in the survey differ is in the **cost to your household**.

Assume that the annual costs to your household would be collected via income taxes over the next 20 years.

Assume that the costs specified in each investment option are used entirely for species recovery efforts.

For **Channel Darter**, money would be spent on targeted initiatives to **improve freshwater quality** in the areas that are crucial for Channel Darter survival.

For **Pugnose Shiner**, money would be spent on **securing and rehabilitating coastal wetlands** that provide habitat necessary for Pugnose Shiner recovery.

For **Lake Sturgeon**, money would be spent on the variety of measures outlined in the strategies above (**fisheries management, restocking, habitat rehabilitation, dam removal**).

Trade-Offs

Previous surveys that have been completed on people's choices concerning paying for government programs usually encounter a difficulty.

- Most do not view the question in terms of actual behaviour.
- They say that they will act one way but actually do something else if the situation were presented to them in real life.

Most people say that they are willing to pay a higher price than they would actually pay if the money was actually taken from them in higher taxes.

- Many scientists believe that this is because respondents do not consider how that money would actually impact a household budget.
- It is easy to be generous when no real money is asked for.

Also, the government has limited funds but still must protect all species at risk (click here **[INSERT HYPERLINK TO 'SARA Ontario POP-UP' – ENSURE IT OPENS IN A NEW WINDOW]** to see the list of species in Ontario that are already protected under SARA or are under consideration for addition to SARA).

- By protecting Pugnose Shiner, for example, a different species in need of protection may not receive all the money needed.
- A trade-off is therefore being made.

Every additional species at risk requires a new recovery plan and more money to be spent.

- Please understand that whatever investment option you choose, the money will not be able to be spent on another species, or on other government priorities such as the economy or health care, unless more money is obtained through taxes.
- You will be making a trade-off by paying for the protection of southern Ontario aquatic species at risk.

Trade-Offs

The following screens will present **two** SARA investment options, along with a no-investment option, that vary according to the characteristics that were just outlined. **You will be asked which of the two options you would most prefer**, given the anticipated long-term impacts on aquatic species at risk in Ontario and the increased cost to your household.

Make your choices as if you really had to choose one of these investment options today.

- That is, if you select an investment option with an annual cost of $10 per year, you are choosing for your annual taxes to be $10 higher for the next 20 years in order to fund this option.
- If you would prefer to not have any increase in taxes and see no new investments in aquatic species recovery efforts, choose the "no investment" option.

Remember, these comparisons are hypothetical and are generated by computer (we want to test all possible combinations from the very best to the very worst). **Assume that all hypothetical combinations are possible** and make your choices accordingly.

[NEW SCREEN]

You will now be shown 8 sets of investment options

- Choose ONLY ONE OPTION on each screen.
- Consider the two options you are shown on EACH SCREEN are the ONLY ones available.
- Each time, please make your choice **independently** from your previous choices **–** do not compare options on different screens.

PROGRAMMER NOTES:

- **RANDOMLY ASSIGN RESPONDENT TO BLOCK 1, BLOCK 2, BLOCK 3, BLOCK 4 OR BLOCK 5**
- **RANDOMIZE THE ORDER OF THE 8 CHIOICE SITUATIONS WITHIN EACH BLOCK**
- **FOR THE DATA FILE, LABEL THE CHOICE SITUATION VARIABLES CS 1 TO 40**
- **CREATE A VARIABLE THAT CAPTURES THE ORDER OF PRESENTATION OF EACH CS [CS1 ORDER, CS2 ORDER, ETC.]**

INVESTMENT CHOICE 1

Please carefully compare the options presented in the table below.

[INSERT TABLE]

Please click on the following links to review information about the Channel Darter, Pugnose Shiner, Lake Sturgeon or Lake Sturgeon recovery strategies. **[INSERT HYPERLINKS TO PREVIOUS INFORMATION SCREENS. ENSURE INFORMATION OPENS IN A NEW WINDOW.]**

V1. If you had to select one of these options, which one would you choose?

PLEASE SELECT ONE RESPONSE ONLY

Investment Option A
Investment Option B
No Investment

INVESTMENT CHOICE 2

Please carefully compare the options presented in the table below.

[INSERT TABLE]

Please click on the following links to review information about the Channel Darter, Pugnose Shiner, Lake Sturgeon or Lake Sturgeon recovery strategies. **[INSERT HYPERLINKS TO PREVIOUS INFORMATION SCREENS. ENSURE INFORMATION OPENS IN A NEW WINDOW.]**

V2. If you had to select one of these options, which one would you choose?

PLEASE SELECT ONE RESPONSE ONLY

Investment Option A
Investment Option B
No Investment

INVESTMENT CHOICE 3

Please carefully compare the options presented in the table below.

[INSERT TABLE]

Please click on the following links to review information about the Channel Darter, Pugnose Shiner, Lake Sturgeon or Lake Sturgeon recovery strategies. **[INSERT HYPERLINKS TO PREVIOUS INFORMATION SCREENS. ENSURE INFORMATION OPENS IN A NEW WINDOW.]**

V3. If you had to select one of these options, which one would you choose?

PLEASE SELECT ONE RESPONSE ONLY

Investment Option A
Investment Option B
No Investment

INVESTMENT CHOICE 4

Please carefully compare the options presented in the table below.

[INSERT TABLE]

Please click on the following links to review information about the Channel Darter, Pugnose Shiner, Lake Sturgeon or Lake Sturgeon recovery strategies. **[INSERT HYPERLINKS TO PREVIOUS INFORMATION SCREENS. ENSURE INFORMATION OPENS IN A NEW WINDOW.]**

V4. If you had to select one of these options, which one would you choose?

PLEASE SELECT ONE RESPONSE ONLY

Investment Option A
Investment Option B
No Investment

INVESTMENT CHOICE 5

Please carefully compare the options presented in the table below.

[INSERT TABLE]

Please click on the following links to review information about the Channel Darter, Pugnose Shiner, Lake Sturgeon or Lake Sturgeon recovery strategies. **[INSERT HYPERLINKS TO PREVIOUS INFORMATION SCREENS. ENSURE INFORMATION OPENS IN A NEW WINDOW.]**

V5. If you had to select one of these options, which one would you choose?

PLEASE SELECT ONE RESPONSE ONLY

Investment Option A
Investment Option B
No Investment

INVESTMENT CHOICE 6

Please carefully compare the options presented in the table below.

[INSERT TABLE]

Please click on the following links to review information about the Channel Darter, Pugnose Shiner, Lake Sturgeon or Lake Sturgeon recovery strategies. **[INSERT HYPERLINKS TO PREVIOUS INFORMATION SCREENS. ENSURE INFORMATION OPENS IN A NEW WINDOW.]**

V6. If you had to select one of these options, which one would you choose?

PLEASE SELECT ONE RESPONSE ONLY

Investment Option A
Investment Option B
No Investment

INVESTMENT CHOICE 7

Please carefully compare the options presented in the table below.

[INSERT TABLE]

Please click on the following links to review information about the Channel Darter, Pugnose Shiner, Lake Sturgeon or Lake Sturgeon recovery strategies. **[INSERT HYPERLINKS TO PREVIOUS INFORMATION SCREENS. ENSURE INFORMATION OPENS IN A NEW WINDOW.]**

V7. If you had to select one of these options, which one would you choose?

PLEASE SELECT ONE RESPONSE ONLY

Investment Option A
Investment Option B
No Investment

INVESTMENT CHOICE 8

Please carefully compare the options presented in the table below.

[INSERT TABLE]

Please click on the following links to review information about the Channel Darter, Pugnose Shiner, Lake Sturgeon or Lake Sturgeon recovery strategies. **[INSERT HYPERLINKS TO PREVIOUS INFORMATION SCREENS. ENSURE INFORMATION OPENS IN A NEW WINDOW.]**

V8. If you had to select one of these options, which one would you choose?

PLEASE SELECT ONE RESPONSE ONLY

Investment Option A
Investment Option B
No Investment

SARA Investment Choices

You have now finished the comparisons. Thanks very much for your careful consideration.

Reasons for Not Choosing SARA Investments

[ASK Q5A IF 'NO INVESTMENT' SELECTED FOR ALL OF THE CHOICE SITUATIONS]

Reasons for Not Choosing SARA Investments

5. A. You indicated you would not choose any of the SARA investment options. Could you please tell us why?

*Please select **all** reasons that factored into your decision making process at the time.*

[RANDOMIZE ORDER EXCEPT FOR OTHER AND DK]
The cost was too high for the benefits received
I can't afford to pay any extra now even if there were long-term benefits for everyone
I do not believe that the program would actually work to increase population numbers
I do not feel it is my responsibility to pay to protect a species at risk
Protecting species is not a priority for me
I don't want more tax added on to what I currently pay
I do not trust the government to effectively run the program
I need more information before I can make this choice
There are better ways to spend public funds
Other (Please specify)
Don't know

[IF DON'T KNOW TO Q5A, SKIP TO Q7. IF ONLY ONE RESPONSE SELECTED AT Q5A, AUTOFILL Q5B AND SKIP TO Q7. ALL OTHERS CONITNUE WITH Q5B]

Reasons for Not Choosing SARA Investments

5B. What was the **most important** reason you did not select any of the SARA investment options?

Please select one response only

[INSERT ITEMS SELECTED IN Q5A IN THE SAME ORDER OF PRESENTATION]
Don't know

Reasons for Choosing SARA Investments

[ASK Q6A & B IF INVESTMENT OPTION A OR INVESTMENT OPTION B SELECTED FOR ANY OF THE CHOICE SITUATIONS]

Reasons for Choosing SARA Investments

6. A. You selected one of the two SARA investment options on at least one occasion. Could you please tell us why?

*Please select **all** reasons that factored into your decision making process at the time.*

[RANDOMIZE ORDER EXCEPT FOR OTHER AND DK]
This is a small amount to pay for the benefits received
I feel that a species at risk should be protected at any cost
It is important to ensure the continuation of the cultural, historical, and environmental significance embodied in that species
It is important to protect the species so that future generations may also enjoy that species
I am more concerned with the overall ecosystem benefits of saving the species rather than the species itself
Other (Please specify)
Don't know

[IF DON'T KNOW TO Q6A, SKIP TO Q7. IF ONLY ONE RESPONSE SELECTED AT Q6A, AUTOFILL Q6B AND SKIP TO Q7. ALL OTHERS CONITNUE WITH Q6B]

Reasons for Choosing SARA Investments

6B. What was the **most important** reason you selected one of the SARA investment options?

Please select one response only

[INSERT ITEMS SELECTED IN Q6A IN THE SAME ORDER OF PRESENTATION]
Don't know

Activity Profile

7. Which of the following activities do you participate in?

Please select all that apply

Swimming/beach activities
Hiking
Canoeing/kayaking/rafting/sailing
Power boating
Skiing
Snowmobiling
Bird watching
Fishing
Wildlife viewing
Mountain biking
Hunting
Photographing nature
Ecotourism
Whale watching
ATVing or dirt biking
Camping
None of the above
Prefer not to answer

8. To which of the following types of organizations do you belong?

Please select all that apply

Fishing or hunting club
Natural history or bird watching club
Other environmental or conservation organization
Outdoor recreation or fitness club
None of the above
Prefer not to answer

Demographics

The final few questions are for statistical calculations. Please be assured all information will be kept completely confidential.

9. For how many years have you lived in Canada?

Please select one response only

Born and raised
More than 20 years
11 to 20 years
6 to 10 years
3 to 5 years
1 or 2 years
Less than one year
Prefer not to answer

[IF BORN AND RAISED IN CANADA OR DECLINE TO RESPOND IN Q9, SKIP TO Q11]

10. How old were you when you left your country of birth?

Please select one response only

Under the age of 12
12 to 17
18 or older
Prefer not to answer

11. As you know, we all live in Canada, but our ancestors come from many different ethnic backgrounds. What is the **main** ethnic background of your ancestors?

Please select one response only

South Asian (from India, Pakistan, Sri Lanka, Bangladesh, or other)
Southeast Asian (from Philippines, Vietnam, Malaysia, Indonesia, Cambodia or other)
East Asian (from China, Hong Kong, Korea, Japan or other)
West Asian or Middle Eastern (from Iran, Afghanistan, Iraq, Lebanon, Israel, Saudi Arabia, United Arab Emirates, Syria, Kazakhstan, or other)
Northern European (from the United Kingdom, Ireland or Scandinavia)
Southern European (from Italy, Greece, Portugal, Spain, Albania, Croatia, Bosnia, Serbia, or other)
Western European (from Germany, Netherlands, Austria, France, Belgium, or other)
Eastern European (from Poland, Romania, former Soviet Republics, Hungary, Czech Republic, Slovakia, or other))
African
Central or South American (from Mexico, El Salvador, Guatemala, Guyana, Colombia, Argentina, Brazil, or other)
Caribbean (from Jamaica, Trinidad and Tobago, Barbados, Granada, or other)

Canadian
Aboriginal/First Nations/Métis
Other (Please specify)
Prefer not to answer

12. Which of the following best describes where you live?

Please select one response only

Acreage, ranch or farm
Town of less than 10,000 people
City with 10,000 to 50,000 people
City of more than 50,000 people
Prefer not to answer

13. What is the highest level of education you have attained?

Please select one response only

Grade school or some high school
High school diploma
Post-secondary technical school
Some college or university
College degree or diploma
University undergraduate degree
University graduate degree
Prefer not to answer

14. Which of the following best describes your employment status?

Please select all that apply

Working full time (35 hours a week or more)
Working part time (less than 35 hours a week)
Student
Homemaker
Retired
Unemployed
Other
Prefer not to answer

15. How many people aged 18 years of age or older contributed to your total household income in 2010?

Please select one response only

One
Two
Three
Four
Five
Six or more
Prefer not to answer

[SURVEY CONSIDERED COMPLETE]

16. What are the first three digits of your postal code of your residential address?

*Please enter in **letter number letter** format with no spaces*

TEXT BOX **[ENSURE INPUT IS ALPHA-NUMERIC-ALPHA FORMAT]**
Prefer not to answer

17. Do you have any other comments about this survey or the *Species at Risk Act* that you would like to share with us? If so, please use the space below.

[VERBATIM RESPONSE]

You've now finished the survey - thanks very much for your help!
