# Supplementary material for: Non-use Economic Values for Little-Known Aquatic Species at Risk: Comparing Choice Experiment Results from Surveys Focused on Species, Guilds, and Ecosystems
Source: Environ Manage. 2016 Jun 13;58:476–90. doi: 10.1007/s00267-016-0716-0 (PMC4969348; doi:10.1007/s00267-016-0716-0)
Supplement: Supplementary file 3 — Supplementary material 3 (PDF 465 kb).S3. Example (pdf) of a full survey for ecosystems [file 267_2016_716_MOESM3_ESM.pdf]

Supplementary Material S3: Example of a full survey for ecosystems

Article title: Non-use economic values for little-known aquatic species at risk: comparing choice experiment results from surveys focused on species, guilds and ecosystems
Journal name: Environmental Management
Authors: Murray A. Rudd[1], Sheri Andres[2]*, Mary Kilfoil[3,4]

[1]Department of Environmental Sciences, Emory University, Atlanta, USA
[2]Policy and Economics – Central and Arctic Region, Fisheries and Oceans Canada, Winnipeg, Manitoba, Canada
[3]Gardner Pinfold Consulting Economists Ltd., Halifax, Nova Scotia, Canada
[4]Present address: Rowe School of Business, Faculty of Management, Dalhousie University, Halifax, Nova Scotia, Canada
* Corresponding author: Sheri.Andres@dfo-mpo.gc.ca

DEPARTMENT OF FISHERIES AND OCEANS
Conservation of Aquatic Species at Risk in Ontario – Ecosystem Survey
FINAL: February 8, 2011

Introduction at Site

[INSERT STANDARD PANEL INTRODUCTION]

Screening

S1. What is your age?

Month / Year of birth [NUMERIC FIELD]

[TRACK AGE QUOTAS BASED ON S1]

S2. What is your gender?

Please select one response only

Male
Female

[TRACK GENDER QUOTAS BASED ON S2]

S3. In what country do you live?

Please select one response only

USA
Canada
Australia
United Kingdom
Other

[CONTINUE IF CANADA, ELSE THANK & TERMINATE]

S4. In which of the following provinces do you reside?

Please select one response only

Newfoundland and Labrador
Prince Edward Island
Nova Scotia
New Brunswick
Quebec
Ontario
Manitoba
Saskatchewan
Alberta
British Columbia
Yukon Territory
Northwest Territories
Nunavut

[CONTINUE IF ONTARIO, ELSE THANK & TERMINATE]

S5. ONTARIO REGION [DO NOT ASK – AUTOFILL FROM PANEL INFO]

GTA
Southwest Ontario
Central Ontario
Eastern Ontario
Northern Ontario

[TRACK REGION QUOTAS BASED ON S5]

S6. What was the total income for all members of your household before taxes in 2010?

Please select one response only

Less than $20 000
$20,000 to $39,999
$40,000 to $59,999
$60,000 to $79,999
$80,000 to $99,999
$100,000 to $124,999
$125,000 to $149,999
$150,000 or more

[TRACK INCOME QUOTAS BASED ON S6]

PANEL DEMOGRAPHICS [APPEND THE FOLLOWING PANEL INFORMATION]

Age
Gender
FSA

Ontario Region
CSD (census subdivision) Name
CMA/CA (Census Metropolitan Area/Census Agglomeration)
Household Income
Education
Employment Status
Occupation (primary panellist)
Own or Rent
Household Size
Number of Children in the Household
Marital Status (primary panellist)

[THIS INFORMATION IS REQUIRED FOR: NON-RESPONDERS, DQs/OVER QUOTA, PARTIAL COMPLETES AND COMPLETES]

Conservation of Aquatic Species at Risk in Ontario

Fisheries and Oceans Canada   Pêches et Océans Canada

Canada

Welcome!

The purpose of this survey is to help government decision-makers better understand the priorities of citizens with regard to the conservation of aquatic (fish, molluscs, reptiles) species at risk in Ontario.

Your participation in this survey is **voluntary** and you may decide to stop participating in the survey at any time. **The information that you provide is important!** We very much appreciate the time and effort you take to complete this survey.

Your answers to the survey will be kept private. Any reports about this study will not identify you in any way. Results will be shown in group form only. None of the personal identifying information you provided to Ipsos when you joined the i-Say panel will be shared with any other individual, organization, or government agency.

Government Priorities

Government Priorities

1. The Government of Canada spends money in a wide variety of areas that directly or indirectly help improve the quality of lives of Canadians. Personal income taxes provide over 49% of the funds used to pay for these services and activities (click here for more information). **[ENSURE THE HYPERLINK OPENS AS A NEW WINDOW]**

In your opinion, how important is it for the Government of Canada to invest in each of the following factors? Please use a scale of 1 to 5 where **1** means **not at all important** and **5** means **very important**.

Please select one response for each item

[ACROSS TOP OF GRID]
1 – Not at all important
2
3
4
5 – Very important

[DOWN SIDE OF GRID] [RANDOMIZE ORDER]
Maintain and build public infrastructure in Canada
Increase overall economic activity (GDP) in Canada
Reduce poverty and inequalities in wealth within Canada
Participate in efforts to reduce conflict and enhance security in Canada and abroad
Develop an active and vibrant arts and culture sector in Canada
Improve the physical and mental health of Canadians
Increase employment opportunities for Canadians
Increase the number of Canadians graduating from high schools, colleges and universities
Build social cohesion and trust in Canadian society
Protect our environment, ecosystems, and biodiversity

The *Species at Risk Act* (SARA)

A number of aquatic species in Canada are listed as species at risk of extinction. Species at risk are protected under the *Species at Risk Act* (SARA) (2003). SARA protects not only the species themselves but also their habitat. According to this Act, a species becomes more at risk as it passes from **Not At Risk** to **Special Concern** to **Threatened** to **Endangered**.

The figure below defines each of the terms used in SARA.

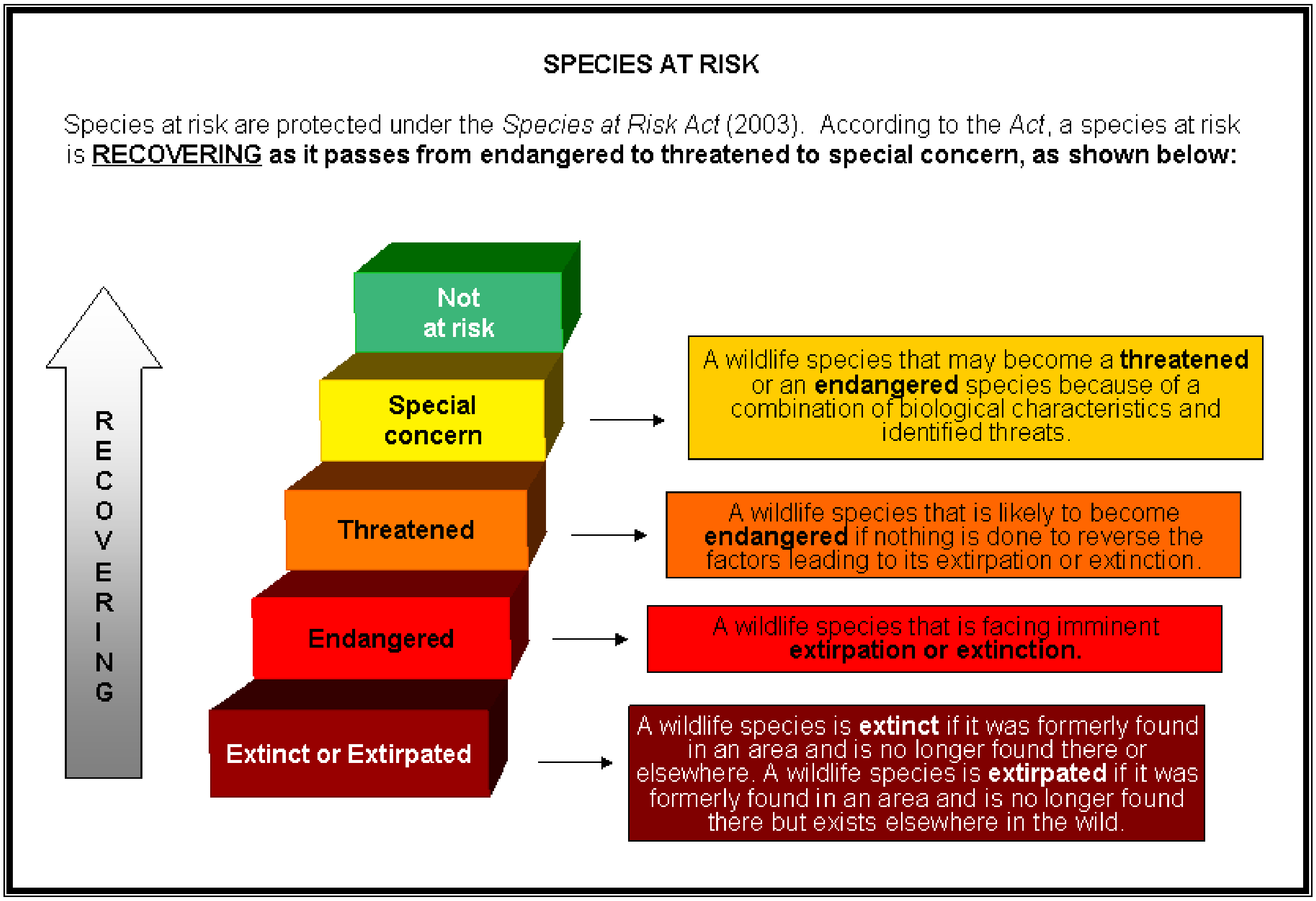

Click <u>here</u> **[INSERT LINK TO 'SARA Ontario POP-UP' – ENSURE IT OPENS AS A NEW WINDOW.]** for a list of species in Ontario that are already protected under SARA or are under consideration for addition to SARA.

2. Before starting this survey, how familiar were you with the ***Species at Risk Act* (SARA)**?

Please select one response only

Very familiar
Somewhat familiar
Not familiar

Threats to Aquatic Species in Ontario

3. A number of factors could threaten various aquatic species in Ontario. **In your opinion, how important are each of the following potential threats to aquatic species in Ontario?** Please use a scale of 1 to 5 where **1** means **not at all important** and **5** means **very important**.

Please select one response for each item

[ACROSS TOP OF GRID]
1 – Not at all important
2
3
4
5 – Very important
Don't know

[DOWN SIDE OF GRID] [RANDOMIZE ORDER]
Water pollution
Recreational fishing
Competition from non-native (invasive) species (e.g. zebra mussels)
Acid rain
Aquaculture (fish farming)
Commercial fishing
Climate change
Habitat loss/degradation from agricultural activities
Habitat loss/degradation from urbanization
Habitat loss/degradation from industrial activities (e.g. oil and gas, forestry and mining exploration/extraction)
Habitat loss/degradation, habitat fragmentation and species mortality/injury from dams and other barriers in rivers and streams

Threats to Aquatic Species in Ontario

4. Are there **other factors** that you think pose a threat to aquatic species in Canada?

Please be detailed and specific in your response. You may enter 'No' or 'Don't know.'

[VERBATIM RESPONSE]

Species Recovery Investment Preferences

[PROGRAMMER NOTE: ENSURE ALL HYPERLINKS OPEN IN A NEW WINDOW]

In the next part of this survey, we are going to show you a series of 8 questions in which you **choose your preferred environmental investment option from two hypothetical alternatives, plus the option to not invest**.

The Southern Ontario investment options include:
(1) improving water quality in streams and rivers; and,
(2) buying and restoring Great Lakes coastal wetlands.

These investments may vary in their impacts on a wide range of aquatic species at risk and other non-threatened species.

The first potential impact of the environmental improvement investment is on the **Water Quality Index (WQI)** in freshwater rivers and streams. Water quality is important for a variety of aquatic species at risk (e.g. Channel Darter; Eastern Sand Darter; and Spotted Sucker). Improvements in the WQI would also have other impacts beyond species at risk recovery.

The second impact of the recovery investment is on the area of **coastal habitat available for wetlands-oriented aquatic species at risk** (e.g., Pugnose Shiner; Lake Chubsucker; Spotted Gar; Pugnose Minnow; and Warmouth). Again, improvements in the quantity and quality of coastal wetlands also have other broad impacts.

The annual cost of the program to your household is also included in the choices.

In the questions that follow, we will ask you to indicate which of two recovery investment options, if either, **you would be willing to pay for**.

The following pages will present more information about how the recovery investment options vary.

Water Quality Index Background Information

[PROGRAMMER NOTE: ENSURE ALL HYPERLINKS OPEN IN A NEW WINDOW]

This indicator, as a water quality index based on many chemical parameters, assesses surface freshwater quality with respect to protecting aquatic life (e.g., fish, invertebrates and plants). It provides a sensitive measure of the health of aquatic ecosystems.

As water quality improves and provides improved conditions under which the health of aquatic life improves, we can expect that there will be a corresponding increase in the abundance of a variety of freshwater species at risk that are sensitive to water quality (e.g. Channel Darter; Eastern Sand Darter; and Spotted Sucker).

The **freshwater Water Quality Index (WQI)**, developed for the Canadian Council of Ministers of the Environment, provides an overall measure of the suitability of water bodies to support aquatic life at selected monitoring sites in Canada. In its final form, the WQI provides a measure that ranges between 0 and 100. The values of the WQI can be interpreted as follows:

Index Value	Interpretation
Excellent (95.0 to 100.0)	Water quality measurements never or very rarely exceed chemical parameters in water quality guidelines
Good (80.0 to 94.9)	Water quality measurements rarely exceed chemical parameters in water quality guidelines. In cases that they do it is by a narrow margin
Fair (65.0 to 79.9)	Water quality measurements sometimes exceed chemical parameters in water quality guidelines, at times by a wide margin
Marginal (45.0 to 64.9)	Water quality measurements often exceed chemical parameters in water quality guidelines and/or do so by a considerable margin
Poor (0 to 44.9)	Water quality measurements usually exceed water quality guidelines and/or do so by a considerable margin.

Water Quality Index Background Information

If the WQI improves, populations of aquatic species at risk may stabilize or increase in their native areas. Other freshwater fish that are not at risk would also benefit from WQI improvement, as would a variety of birds, molluscs, plants, and terrestrial animals.

Freshwater quality improvement measures could include:
- establishing riparian buffers (vegetated areas bordering and protecting water bodies);
- water management; and,
- improvements in wastewater treatment.

Click here for more information.

[INFORMATION FOR POP-UP – ENSURE IT OPENS IN A NEW WINDOW]
Freshwater quality improvement measures

Traditional agricultural practices can have a negative impact on water quality. There are numerous best management or mitigation measures which could be used to improve water quality.

- One of the most important practices is the establishment of a riparian buffer (a vegetated area that borders a body of water which helps shade and protect the water body from the impact of adjacent land uses). Stable riverbanks decrease turbidity (a measure of the degree to which the water loses its transparency due to the presence of suspended particles) and sediment loading (the solid material that is transported by a natural agent, especially by a stream) which is beneficial for many fish species. The vegetation also traps nutrients and contaminants such as pesticides and decreases their introduction into the river system. This can be achieved in a number of ways - for example, fencing along a riverbank keeps livestock from the river. In areas farmed for crops, leaving an unplowed area may be all that is required.

- A second important practice is water management. Water that is drained from the land quickly can take with it higher levels of contaminants. Ensuring the water stays on the land, in water management ponds or wetlands can also improve water quality.

- Wastewater treatment facility outflow can also have a negative impact on water quality. New wastewater treatment facilities can be quite expensive, but are not always necessary. Performance evaluation of existing operational activities can result in substantial cost savings with an improvement in water quality.

[NEW SCREEN]

Riverine Fish Species

Freshwater quality improvement measures such as the ones described above would play a particularly important role for riverine species recovery. The species pictured below are examples of riverine species which may benefit:

(insert image- fish eco) (center on page)

Photo credits:Eastern Sand Darter – Al Dextrase, Ontario Ministry of Natural Resources; Channel Darter – G. Coker; Spotted Sucker – Konrad Schmidt

Water Quality Index Background Information

Freshwater quality is **currently** rated as "good" or "excellent" at 60% of southern Ontario sites, "fair" at 30% and "marginal" or "poor" at 10%.

We assume that **somewhat improved** water quality would result in:
- 70% of sites being good or excellent
- 24% of sites being fair
- 6% being marginal or poor.

A **much improved** situation would result in:
- 78% of sites being good or excellent
- 18% of sites being fair
- 4% of sites being marginal or poor

Freshwater Quality:
Water Quality Index (WQI) Ratings for Sites in Southern Ontario

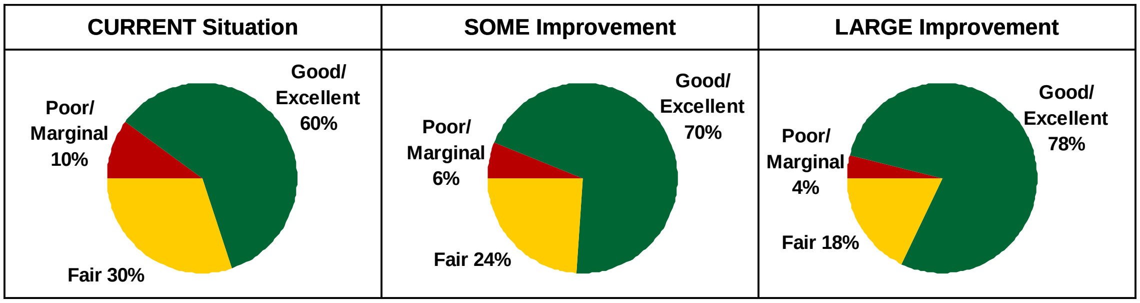

Coastal Wetlands Background Information

Coastal lakeshore and wetland habitat is extremely important for species that rely on coastal wetlands as their primary habitat. This group includes Pugnose Shiner, Lake Chubsucker, Spotted Gar, Pugnose Minnow, and Warmouth. **[ENSURE ALL HYPERLINKS OPEN IN A NEW WINDOW]**

(eco fish 2—insert image)

Photo credits: Pugnose Shiner, Lake Chubsucker, Pugnose Minnow and Warmouth - Konrad Schmidt; Spotted Gar – Jason Barnusz, Fisheries and Oceans Canada

Declines of these fishes have been attributed to their sensitivity to decreases in water clarity, loss of habitat from shoreline development, and loss of native aquatic vegetation.

Populations of these types of fishes may stabilize or increase when suitable habitat is available. **Coastal wetland rehabilitation programs**, such as:

- wetland purchase
- wetland preservation
- wetland rehabilitation

would play a particularly important role for the recovery of these five species.

Click here for more information on wetlands purchase, preservation and rehabilitation. **[ENSURE HYPERLINK OPENS IN A NEW WINDOW]**

Click here for more information on Great Lakes Coastal Wetlands - Science and Conservation**. [ENSURE HYPERLINK OPENS IN A NEW WINDOW]**

[POP-UP INFORMATION]
Information on wetlands purchase, preservation and rehabilitation

Wetlands purchase can include acquiring land through the following methods:

- outright purchase
- land donation
- conservation agreement (a legal agreement in which a landowner agrees to the imposition of restrictions on activities that would threaten the ecological value of the land.)
- relinquishment of land use rights (relinquishment of land use rights (for example mineral or timber rights) held by a private entity, to enable publicly-held land or water to be designated as a protected area.)

Wetlands preservation can include the following actions:

- developing property management plans to document the condition of the land and identify priority actions needed to protect key species and habitats over the long term
- monitoring properties on a regular basis to certify that key natural features remain protected.
- responding to unforeseen threats or issues as they may arise.

Wetlands rehabilitation can include:

- hydrological rehabilitation: re-establishing natural water level variability which may include the use of dykes or new channels for directing water through the wetland
- biological rehabilitation: altering existing habitat to encourage the re-establishment of desirable plants and animals
- chemical or contaminant reduction: reduction at the source (e.g. decreasing fertilizer application to agricultural lands or reducing discharge from a sewage treatment plant) or, in cases where a point source cannot be located, rehabilitation in the wetlands (e.g. capping or carefully removing contaminated sediments).

Wetlands also provide other benefits to humans, including:

- filtration of water,
- flood retention,
- erosion reduction,
- recreation opportunities (canoeing, fishing, bird watching),
- harvesting (berries, grains),
- carbon storage,
- nutrient cycling, and
- groundwater recharge

Coastal Wetlands Background Information

The Mixed Wood Plains ecozone encompasses the lower Great Lakes (Ontario and Erie) and the St. Lawrence Valley and is home to half of Canada's population.

According to a recent report[1] (click here to access the full report) **wetlands accounted for 25% of the land area in this ecozone** prior to European settlement.

By 2002 wetlands accounted for **only 6.9%** of this land area.

If the recent rate of reduction continued, by 2011 wetlands would account for **only 6.5%** of this area.

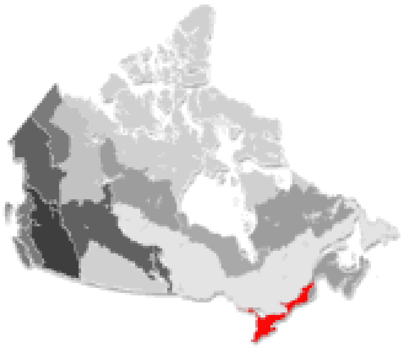

Map: Natural Resources Canada

We assume that SOMEWHAT improved wetlands cover would result in 7% (or 572,000 hectares) of total wetland cover and a MUCH improved situation would result in 8% (or 654,000 hectares) of total wetland cover.

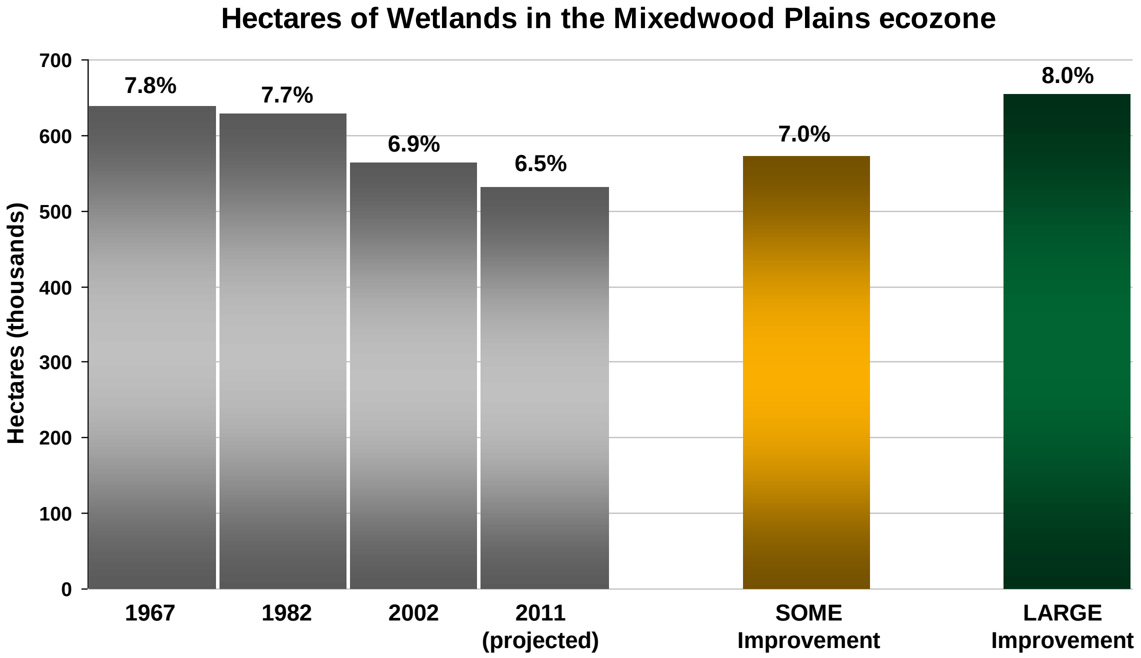

[1] This study only considered large wetlands (>10 hectares). Smaller wetlands (< 10 hectares) contribute to a relatively large proportion of total wetland area in this ecozone – if these smaller wetlands had been considered in the study, the total wetlands area would be greater but the annual loss would be even more significant.

Program Cost

So far, we have described the potential environmental benefits arising from new SARA recovery investments. There is, of course, a need to pay for these investments so the final way that the options in the survey differ is in the **cost to your household**.

Assume that the annual costs to your household would be collected via income taxes over the next 20 years.

Assume that the costs specified in each investment option are used entirely for species recovery efforts. Investments would include targeted initiatives to **improve freshwater quality** in the areas that are crucial for survival of these aquatic species at risk and on **securing and rehabilitating coastal wetlands** that provide habitat necessary for fish population recovery.

Trade-Offs

Previous surveys that have been completed on people's choices concerning paying for government programs usually encounter a difficulty.

- Most do not view the question in terms of actual behaviour.
- They say that they will act one way but actually do something else if the situation were presented to them in real life.

Most people say that they are willing to pay a higher price than they would actually pay if the money was actually taken from them in higher taxes.

- Many scientists believe that this is because respondents do not consider how that money would actually impact a household budget.
- It is easy to be generous when no real money is asked for.

Also, the government has limited funds but still must protect all species at risk (click here **[INSERT HYPERLINK TO 'SARA Ontario POP-UP' – ENSURE IT OPENS IN A NEW WINDOW]** to see the list of species in Ontario that are already protected under SARA or are under consideration for addition to SARA).

- By protecting Pugnose Shiner, for example, a different species in need of protection may not receive all the money needed.
- A trade-off is therefore being made.

Every additional species at risk requires a new recovery plan and more money to be spent.

- Please understand that whatever investment option you choose, the money will not be able to be spent on another species, or on other government priorities such as the economy or health care, unless more money is obtained through taxes.
- You will be making a trade-off by paying for the protection of southern Ontario aquatic species at risk.

Trade-Offs

The following screens will present **two** SARA investment options, along with a no-investment option, that vary according to the characteristics that were just outlined. **You will be asked which of the two options you would most prefer**, given the anticipated long-term impacts on aquatic species at risk in Ontario and the increased cost to your household.

Make your choices as if you really had to choose one of these investment options today.

- That is, if you select an investment option with an annual cost of $10 per year, you are choosing for your annual taxes to be $10 higher for the next 20 years in order to fund this option.
- If you would prefer to not have any increase in taxes and see no new investments in aquatic species recovery efforts, choose the "no investment" option.

Remember, these comparisons are hypothetical and are generated by computer (we want to test all possible combinations from the very best to the very worst). **Assume that all hypothetical combinations are possible** and make your choices accordingly.

[NEW SCREEN]

The investments presented may result in **no change**, **some improvement** or a **large improvement** to the **Freshwater Water Quality Index** (WQI) or the **Coastal Wetlands**.

	RECOVERING →				
SARA LEVELS	Extirpated	Endangered	Threatened	Special Concern	Not at Risk

Freshwater Water Quality Index (WQI)

	No Change in WQI	Some Improvement in WQI	Large Improvement in WQI
	At least TWO species at risk DEGRADE by one level	At least TWO species at risk IMPROVE by one level	At least FOUR species at risk IMPROVE by one level, or at least TWO species at risk IMPROVE by two levels
	No species at risk IMPROVE	No species at risk DECLINE	No species at risk DECLINE
	Freshwater Water Quality Index (WQI) - % of sites		
Good/excellent	60%	70%	78%
Fair	30%	24%	18%
Poor/marginal	10%	6%	4%

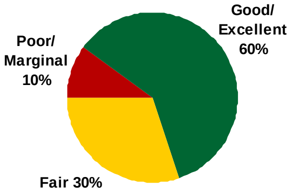

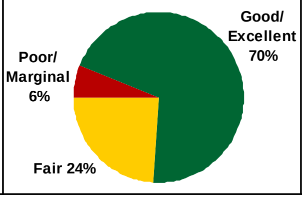

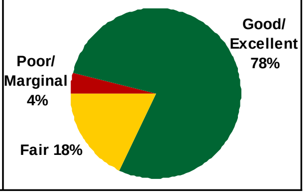

Coastal Wetlands

Current Wetland Habitat Maintained	Some Increase in Wetland Habitat	Large Increase in Wetland Habitat
At least TWO species at risk DEGRADE by one level	At least TWO species at risk IMPROVE by one level	At least FOUR species at risk IMPROVE by one level, or at least TWO species at risk IMPROVE by two levels
No species at risk IMPROVE	No species at risk DECLINE	No species at risk DECLINE
**529,000 hectares**	**572,000 hectares**	**654,000 hectares**
**6.5%**	**7.0%**	**8.0%**

[NEW SCREEN]

You will now be shown 8 sets of investment options

- Choose ONLY ONE OPTION on each screen.
- Consider the two options you are shown on EACH SCREEN are the ONLY ones available.
- Each time, please make your choice **independently** from your previous choices – do not compare options on different screens.

PROGRAMMER NOTES:

- **RANDOMLY ASSIGN RESPONDENT TO BLOCK 1, BLOCK 2 OR BLOCK 3 USING 'LEAST COUNT' METHOD [I.E. SUCH THAT EACH BLOCK IS COMPLETED BY THE SAME NUMBER OF RESPONDENTS]**
- **RANDOMIZE THE ORDER OF THE 8 CHIOICE SITUATIONS WITHIN EACH BLOCK**
- **FOR THE DATA FILE, LABEL THE CHOICE SITUATION VARIABLES CS 1 TO 24**
- **CREATE A VARIABLE THAT CAPTURES THE ORDER OF PRESENTATION OF EACH CS [CS1 ORDER, CS2 ORDER, ETC.]**

INVESTMENT CHOICE 1

Please carefully compare the options presented in the table below.

[INSERT TABLE]

Please click on the following links to review information about the Freshwater Water Quality Index (WQI) or the Coastal Wetlands. **[INSERT HYPERLINKS TO PREVIOUS INFORMATION SCREENS. ENSURE INFORMATION OPENS IN A NEW WINDOW.]**

V1. If you had to select one of these options, which one would you choose?

PLEASE SELECT ONE RESPONSE ONLY

Investment Option A
Investment Option B
No Investment

INVESTMENT CHOICE 2

Please carefully compare the options presented in the table below.

[INSERT TABLE]

Please click on the following links to review information about the Freshwater Water Quality Index (WQI) or the Coastal Wetlands. **[INSERT HYPERLINKS TO PREVIOUS INFORMATION SCREENS. ENSURE INFORMATION OPENS IN A NEW WINDOW.]**

V2. If you had to select one of these options, which one would you choose?

PLEASE SELECT ONE RESPONSE ONLY

Investment Option A
Investment Option B
No Investment

INVESTMENT CHOICE 3

Please carefully compare the options presented in the table below.

[INSERT TABLE]

Please click on the following links to review information about the Freshwater Water Quality Index (WQI) or the Coastal Wetlands. **[INSERT HYPERLINKS TO PREVIOUS INFORMATION SCREENS. ENSURE INFORMATION OPENS IN A NEW WINDOW.]**

V3. If you had to select one of these options, which one would you choose?

PLEASE SELECT ONE RESPONSE ONLY

Investment Option A
Investment Option B
No Investment

INVESTMENT CHOICE 4

Please carefully compare the options presented in the table below.

[INSERT TABLE]

Please click on the following links to review information about the Freshwater Water Quality Index (WQI) or the Coastal Wetlands. **[INSERT HYPERLINKS TO PREVIOUS INFORMATION SCREENS. ENSURE INFORMATION OPENS IN A NEW WINDOW.]**

V4. If you had to select one of these options, which one would you choose?

PLEASE SELECT ONE RESPONSE ONLY

Investment Option A
Investment Option B
No Investment

INVESTMENT CHOICE 5

Please carefully compare the options presented in the table below.

[INSERT TABLE]

Please click on the following links to review information about the Freshwater Water Quality Index (WQI) or the Coastal Wetlands. **[INSERT HYPERLINKS TO PREVIOUS INFORMATION SCREENS. ENSURE INFORMATION OPENS IN A NEW WINDOW.]**

V5. If you had to select one of these options, which one would you choose?

PLEASE SELECT ONE RESPONSE ONLY

Investment Option A
Investment Option B
No Investment

INVESTMENT CHOICE 6

Please carefully compare the options presented in the table below.

[INSERT TABLE]

Please click on the following links to review information about the Freshwater Water Quality Index (WQI) or the Coastal Wetlands. **[INSERT HYPERLINKS TO PREVIOUS INFORMATION SCREENS. ENSURE INFORMATION OPENS IN A NEW WINDOW.]**

V6. If you had to select one of these options, which one would you choose?

PLEASE SELECT ONE RESPONSE ONLY

Investment Option A
Investment Option B
No Investment

INVESTMENT CHOICE 7

Please carefully compare the options presented in the table below.

[INSERT TABLE]

Please click on the following links to review information about the Freshwater Water Quality Index (WQI) or the Coastal Wetlands. **[INSERT HYPERLINKS TO PREVIOUS INFORMATION SCREENS. ENSURE INFORMATION OPENS IN A NEW WINDOW.]**

V7. If you had to select one of these options, which one would you choose?

PLEASE SELECT ONE RESPONSE ONLY

Investment Option A
Investment Option B
No Investment

INVESTMENT CHOICE 8

Please carefully compare the options presented in the table below.

[INSERT TABLE]

Please click on the following links to review information about the Freshwater Water Quality Index (WQI) or the Coastal Wetlands. **[INSERT HYPERLINKS TO PREVIOUS INFORMATION SCREENS. ENSURE INFORMATION OPENS IN A NEW WINDOW.]**

V8. If you had to select one of these options, which one would you choose?

PLEASE SELECT ONE RESPONSE ONLY

Investment Option A
Investment Option B
No Investment

SARA Investment Choices

You have now finished the comparisons. Thanks very much for your careful consideration.

[ASK Q5A IF 'NO INVESTMENT' SELECTED FOR ALL OF THE CHOICE SITUATIONS]

Reasons for Not Choosing SARA Investments

5. A. You indicated you would not choose any of the SARA investment options. Could you please tell us why?

*Please select **all** reasons that factored into your decision making process at the time.*

[RANDOMIZE ORDER EXCEPT FOR OTHER AND DK]
The cost was too high for the benefits received
I can't afford to pay any extra now even if there were long-term benefits for everyone
I do not believe that the program would actually work to increase population numbers
I do not feel it is my responsibility to pay to protect a species at risk
Protecting species is not a priority for me
I don't want more tax added on to what I currently pay
I do not trust the government to effectively run the program
I need more information before I can make this choice
There are better ways to spend public funds
Other (Please specify)
Don't know

[IF DON'T KNOW TO Q5A, SKIP TO Q7. IF ONLY ONE RESPONSE SELECTED AT Q5A, AUTOFILL Q5B AND SKIP TO Q7. ALL OTHERS CONITNUE WITH Q5B]

Reasons for Not Choosing SARA Investments

5B. What was the **most important** reason you did not select any of the SARA investment options?

Please select one response only

[INSERT ITEMS SELECTED IN Q5A IN THE SAME ORDER OF PRESENTATION]
Don't know

[ASK Q6A & B IF INVESTMENT OPTION A OR INVESTMENT OPTION B SELECTED FOR ANY OF THE CHOICE SITUATIONS]

Reasons for Choosing SARA Investments

6. A. You selected one of the two SARA investment options on at least one occasion. Could you please tell us why?

*Please select **all** reasons that factored into your decision making process at the time.*

[RANDOMIZE ORDER EXCEPT FOR OTHER AND DK]
This is a small amount to pay for the benefits received
I feel that a species at risk should be protected at any cost
It is important to ensure the continuation of the cultural, historical, and environmental significance embodied in that species
It is important to protect the species so that future generations may also enjoy that species
I am more concerned with the overall ecosystem benefits of saving the species rather than the species itself
Other (Please specify)
Don't know

[IF DON'T KNOW TO Q6A, SKIP TO Q7. IF ONLY ONE RESPONSE SELECTED AT Q6A, AUTOFILL Q6B AND SKIP TO Q7. ALL OTHERS CONITNUE WITH Q6B]

Reasons for Choosing SARA Investments

6B. What was the **most important** reason you selected one of the SARA investment options?

Please select one response only

[INSERT ITEMS SELECTED IN Q6A IN THE SAME ORDER OF PRESENTATION]
Don't know

Activity Profile

7. Which of the following activities do you participate in?

Please select all that apply

Swimming/beach activities
Hiking
Canoeing/kayaking/rafting/sailing
Power boating
Skiing
Snowmobiling
Bird watching
Fishing
Wildlife viewing
Mountain biking
Hunting
Photographing nature
Ecotourism
Whale watching
ATVing or dirt biking
Camping
None of the above
Prefer not to answer

8. To which of the following types of organizations do you belong?

Please select all that apply

Fishing or hunting club
Natural history or bird watching club
Other environmental or conservation organization
Outdoor recreation or fitness club
None of the above
Prefer not to answer

Demographics

The final few questions are for statistical calculations. Please be assured all information will be kept completely confidential.

9. For how many years have you lived in Canada?

Please select one response only

Born and raised
More than 20 years
11 to 20 years
6 to 10 years
3 to 5 years
1 or 2 years
Less than one year
Prefer not to answer

[IF BORN AND RAISED IN CANADA OR DECLINE TO RESPOND IN Q9, SKIP TO Q11]

10. How old were you when you left your country of birth?

Please select one response only

Under the age of 12
12 to 17
18 or older
Prefer not to answer

11. As you know, we all live in Canada, but our ancestors come from many different ethnic backgrounds. What is the **main** ethnic background of your ancestors?

Please select one response only

South Asian (from India, Pakistan, Sri Lanka, Bangladesh, or other)
Southeast Asian (from Philippines, Vietnam, Malaysia, Indonesia, Cambodia or other)
East Asian (from China, Hong Kong, Korea, Japan or other)
West Asian or Middle Eastern (from Iran, Afghanistan, Iraq, Lebanon, Israel, Saudi Arabia, United Arab Emirates, Syria, Kazakhstan, or other)
Northern European (from the United Kingdom, Ireland or Scandinavia)
Southern European (from Italy, Greece, Portugal, Spain, Albania, Croatia, Bosnia, Serbia, or other)
Western European (from Germany, Netherlands, Austria, France, Belgium, or other)
Eastern European (from Poland, Romania, former Soviet Republics, Hungary, Czech Republic, Slovakia, or other))
African
Central or South American (from Mexico, El Salvador, Guatemala, Guyana, Colombia, Argentina, Brazil, or other)
Caribbean (from Jamaica, Trinidad and Tobago, Barbados, Granada, or other)

Canadian
Aboriginal/First Nations/Métis
Other (Please specify)
Prefer not to answer

12. Which of the following best describes where you live?

Please select one response only

Acreage, ranch or farm
Town of less than 10,000 people
City with 10,000 to 50,000 people
City of more than 50,000 people
Prefer not to answer

13. What is the highest level of education you have attained?

Please select one response only

Grade school or some high school
High school diploma
Post-secondary technical school
Some college or university
College degree or diploma
University undergraduate degree
University graduate degree
Prefer not to answer

14. Which of the following best describes your employment status?

Please select all that apply

Working full time (35 hours a week or more)
Working part time (less than 35 hours a week)
Student
Homemaker
Retired
Unemployed
Other
Prefer not to answer

15. How many people aged 18 years of age or older contributed to your total household income in 2010?

Please select one response only

One
Two
Three
Four
Five
Six or more
Prefer not to answer

[SURVEY CONSIDERED COMPLETE]

16. What are the first three digits of your postal code of your residential address?

*Please enter in **letter number letter** format with no spaces*

TEXT BOX **[ENSURE INPUT IS ALPHA-NUMERIC-ALPHA FORMAT]**
Prefer not to answer

17. Do you have any other comments about this survey or the *Species at Risk Act* that you would like to share with us? If so, please use the space below.

[VERBATIM RESPONSE]

You've now finished the survey - thanks very much for your help!
